# A robust method for the estimation and visualization of IgE cross-reactivity likelihood between allergens belonging to the same protein family

**DOI:** 10.1371/journal.pone.0208276

**Published:** 2018-11-29

**Authors:** Maksymilian Chruszcz, A. Brenda Kapingidza, Coleman Dolamore, Krzysztof Kowal

**Affiliations:** 1 Department of Chemistry and Biochemistry, University of South Carolina, Columbia, South Carolina, United States of America; 2 Department of Allergology and Internal Medicine, Medical University of Bialystok, Bialystok, Poland; 3 Department of Experimental Allergology and Immunology, Medical University of Bialystok, Bialystok, Poland; Mie Daigaku, JAPAN

## Abstract

Among the vast number of identified protein families, allergens emanate from relatively few families which translates to only a small fraction of identified protein families. In allergy diagnostics and immunotherapy, interactions between immunoglobulin E and allergens are crucial because the formation of an allergen-antibody complex is necessary for triggering an allergic reaction. In allergic diseases, there is a phenomenon known as cross-reactivity. Cross-reactivity describes a situation where an individual has produced antibodies against a particular allergenic protein, but said antibodies fail to discriminate between the original sensitizer and other similar proteins that usually belong to the same family. To expound the concept of cross-reactivity, this study examines ten protein families that include allergens selected specifically for the analysis of cross-reactivity. The selected allergen families had at least 13 representative proteins, overall folds that differ significantly between families, and include relevant allergens with various potencies. The selected allergens were analyzed using information on sequence similarities and identities between members of the families as well as reports on clinically relevant cross-reactivities. Based on our analysis, we propose to introduce a new A-RISC index (Allergens’–Relative Identity, Similarity and Cross-reactivity) which describes homology between two allergens belonging to the same protein family and is used to predict the likelihood of cross-reactivity between them. Information on sequence similarities and identities, as well as on the values of the proposed A-RISC index is used to introduce four categories describing a risk of a cross-reactive reaction, namely: high, medium-high, medium-low and low. The proposed approach can facilitate analysis in component-resolved allergy diagnostics, generation of avoidance guidelines for allergic individuals, and help with the design of immunotherapy.

## Introduction

Allergens originate from many different sources and can stimulate the human immune system to produce immunoglobulin E (IgE) antibodies and/or are responsible for eliciting symptoms of allergy in sensitized individuals. Currently, there are over one thousand such molecules identified and registered by the World Health Organization and International Union of Immunological Societies (WHO/IUIS) Allergen Nomenclature Sub-committee [1]. Surprisingly, allergens arise from relatively few protein families [2–5] which represent only a small fraction of the protein families described in the Pfam database [6].

Interactions between IgE and allergens are crucial for allergic diseases, as the formation of an allergen-antibody complex is necessary for triggering an allergic reaction. The IgE-mediated allergic reaction requires an allergen to cross-link the antibodies bound to the high-affinity receptors located on mast cells [7]. Therefore, in molecular allergology there is significant effort directed to understanding interactions between allergens and antibodies [8–11]. For example, such efforts aim to identify IgE binding epitopes and epitope-paratope interactions [8, 12, 13]. Although allergen-antibody interactions may be studied using various methods, structural biology provides one of the most interesting insights into this molecular phenomenon [12, 14–16]. Thanks to advancements of X-ray crystallography and NMR, we can picture epitopes as being relatively small fragments of proteins recognized by antibodies. Structural biology also provides insight on the structures of many allergens [11, 15]. Therefore, in most cases, it is possible through homology modeling to elucidate information on the tertiary structure of these molecules provided that the protein sequence is available. In parallel with the increase in knowledge on allergen structures, there is an astonishing improvement of allergen purification and standardization methodology. This allows for the identification of allergens even in complex mixtures and facilitate characterization of these molecules [17–21]. Moreover, the production of recombinant allergens has become a standard practice, and it has a direct impact on allergy diagnostics and immunotherapy [16, 22–25].

The understanding of antibody allergen interactions allows for reliable estimation of IgE cross-reactivity. Presence of cross-reactivity corresponds to a situation when an individual has antibodies raised against a particular allergenic protein and these antibodies fail to discriminate between the original sensitizer and bind to other, usually structurally similar, proteins. It must be stressed that in this manuscript we focus on cross-reactivity (we assume that there was one sensitizing allergen), and we are not interested in multisensitization, as the multisensitization refers to the generation of unrelated IgE responses [26]. It is also assumed that in the case of allergy we face the polyclonal cross-reactivity [27]. Moreover, we concentrate on cross-reactivity between allergens belonging to the same protein family that share the same overall fold.

In the process to decipher cross-reactivity between allergens, a minimal approach may use just the information on sequence identity for an estimation of a cross-reactivity likelihood; or combine such information with phylogenetic analysis leading to the elucidation of evolutionary relation between allergens that are recognized by the same antibodies. For example, it was proposed that in most cases, cross-reactivity requires more than 70% sequence identity and is rare when the sequence identity falls below 50% [28]. Evidently, these simple rules seem to help in predicting the observed IgE cross-reactivity. Of course, the comparison of allergens, and especially the prediction of allergenicity, can be significantly more complex than desired as there are various dedicated computational tools that allow us to perform such analysis [9, 29–34]. However, here we are introducing a simple approach that uses mainly information on the primary structures of allergens in combination with various assumptions and observations related to their interactions with antibodies. Our approach also takes advantage of the fact that some of the allergens’ families have many identified and characterized members, which in turn allows us to derive more precise guidelines to estimate the likelihood of cross-reactivity between members of the same protein family. We present here a cross-reactivity analysis of ten allergen families using the A-RISC index and contrast our findings with reports on IgE cross-reactivity determined using experimental approaches. Our approach to the analysis of allergens’ sequences not only considers possible interactions with antibodies, but also results in a single numerical value that describes the likelihood of cross-reactivity between two allergens originating from the same family of proteins. Based on our analysis, we propose to introduce four categories describing a risk of a cross-reactive reaction, namely: high, medium-high, medium-low, and low. We believe that this proposed model is a better estimate of possible cross-reactivity for pairs of allergens having low or medium levels of sequence identity. Moreover, the proposed approach may facilitate analysis in component-resolved allergy diagnostics and generation of avoidance guidelines for allergic individuals or help with the design of immunotherapy.

## Materials and methods

### Selection of allergens

Ten protein families that include allergens were selected for the analysis (Table 1, Supplementary S1–S10 Tables). The selected allergen families had at least 13 representative proteins and overall folds that differ significantly, but also include relevant allergens with various potencies. Protein sequences were obtained by searching Allfam [5], Allergome [35] and Allergen Nomenclature [1] databases. Only proteins that had complete sequences were considered. Furthermore, the sequences that were used for further analysis correspond to the mature version of proteins (signal- and pro-peptides were omitted). This study included allergens that are registered by the World Health Organization and International Union of Immunological Societies (WHO/IUIS) Allergen Nomenclature Sub-committee (www.allergen.org) as well as allergens that are listed by Allergome and/or Allfam and not officially registered. In the case of the officially registered allergens, the full nomenclature name which includes four digits after a period in the numerical part of an allergen symbol was used [1]. Many of the allergens selected have several isoallergens reported. For the isoallergens that have the same digit in the second place, only one representative sequence was chosen and, most often, it was the isoallergen with full nomenclature name ending with a 1. This approach is related to the fact that currently the third and fourth digits of the nomenclature name are determining variants of an isoallergen, and the variant usually has more than 90% sequence identity [1]. However, in some cases when a full sequence for such a protein was not available, isoallergens with names having 2 in the fourth place after the period were chosen. The selected sequence of allergens belonging to each family were stored in Fasta format.

**Table 1:** Summary on selected allergen families. A * indicates prolamin superfamily [5], however only nsLTPs are taken into account.

Protein family	Allergen family ID	# of allergens	Average sequence		Range of sequence	
			identity	similarity	identity	similarity
Papain-like cysteine proteases	AF030	15	37%	50%	21–85%	30–88%
NCP2 family (Group 2 mite allergens)	AF111	17	41%	58%	22–90%	34–95%
Serum albumins	AF056	14	71%	80%	43–99%	56–99%
Pectate lyases	AF073	14	64%	70%	44–98%	51–98%
Lipocalins	AF015	29	21%	33%	4–95%	7–98%
β-expansins and expansin-related	AF093	27	56%	67%	24–99%	38–99%
Group V/VI grass pollen allergens	AF102	13	57%	66%	13–82%	23–85%
Profilins	AF051	64	76%	84%	32–99%	43–99%
Non-specific Lipid Transport Proteins (nsLTPs)	AF050*	51	51%	61%	18–99%	25–99%
Bet v 1 family (PR-10s)	AF069	46	52%	65%	15–98%	28–98%

### Analysis of sequences

**Fig 1:**
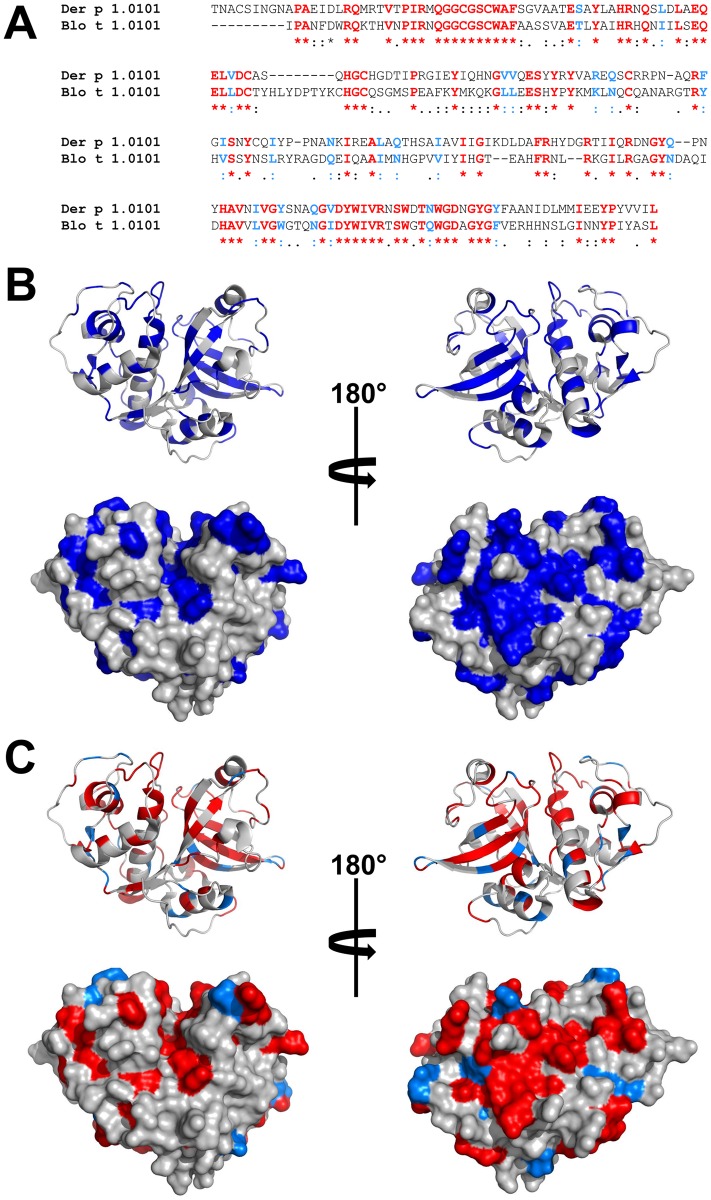
Comparison of Der p 1.0101 and Blot 1.0101. (A) Sequence alignment of Der p 1.0101 and Blo t 1.0101 made with Clustal Omega. Similar residues are marked with bold font type. Similarity definition corresponds to a default setting of the SIAS server. Identical residues are marked in red, while residues marked in light blue correspond to the difference between similar and identical residues. Consensus line corresponds to the similarity definition used by Clustal Omega. It is important to note that the similarity definition used by SIAS is more conservative than the one used by Clustal Omega. (B) Similar amino acids (blue) marked on the structure of Der p 1. The structure is shown in ribbon (top) and space-filling representation (bottom). (C) Identical amino acids (red) mapped on the structure of Der p 1. Residues marked in light blue correspond to the difference between similar and identical residues. We assume that only half of the amino acids marked in light blue (similar, but not identical) will participate in the binding of cross-reactive antibodies. A-RISC index for Der p 1.0101 and Blo t 1.0101pair has a value of 0.37. Figures B and C were generated in Pymol [37].

Multiple sequence alignment of proteins belonging to a single family were performed with Clustal Omega [36]. Sequence similarities and identities were calculated with SIAS (http://imed.med.ucm.es/Tools/sias.html) using the default parameters. The SIAS webpage divides amino acids into the following similarity groups: aromatic (F, Y and W), aliphatic (I, L and V), positively charged (H, K and R), negatively charged (D and E), small with hydroxyl group (S and T) and neutral polar (N and Q). The five remaining amino acids (A, C, G, M and P) are not included to any similarity groups. Sequence similarities and identities calculated with SIAS were stored and used for the generation of various plots as well as for A-RISC calculations (Allergens’–Relative Identity, Similarity and Cross-reactivity) indexes. In this manuscript, an A-RISC index for a particular pair of proteins is defined as an average of the proteins’ sequence similarity (S), as calculated by SIAS server, and identity (I). The index we propose is a single numeric value that provides information on relative homology between allergens from a particular protein family and a selected member of this family. In the case of allergens, a physical meaning of the A-RISC index can be explained by considering interactions of these proteins with antibodies. For example, in a case of epitope and paratope recognition may be mediated by hydrogen bonds, hydrophobic interactions, shape complementarity, etc. The presence of the same or similar amino acids in the corresponding regions of two proteins can lead to a situation whereby the same antibody will fail to discriminate between the proteins, as the paratope will be able to form interactions with the same or very similar epitopes that are present in both proteins (allergens). In this model, we make several assumptions that allow for the simplification of the comparison of two allergens as well as allergen-antibody interactions. For example, we use only information on allergen sequences (primary structure) and ignore secondary and quaternary structures. The only assumption on the tertiary structurers is related to the fact that proteins from the same family generally adopt the same overall fold. Furthermore, we presume that when two protein sequences are compared, we can divide the compared amino acids into three groups: identical, similar, and not similar. We define similar as: “similar” = “identical” + “similar, but not identical”. Additionally, we assume that in the case of two allergens belonging to the same protein family, only identical and similar amino acids are responsible for cross-reactivity. We also make an arbitrary assumption that in the cases of non-identical, but similar amino acids, only in 50% of cases will we observe participation of these residues in binding of a cross-reactive antibody. The previous two assumptions allow us to write the following equation that describes a fraction of all amino acids which may be responsible for interaction with a cross-reactive antibody:

$$I+\;\frac{S-I}{2}\;=\;\frac{I+S}{2},$$ (1)

and provide the formula for calculation of the A-RISC index. An example of the proposed here comparison of two allergens is illustrated in Fig 1. One should note that the difference between A-RISC and sequence identity (expressed as fraction) values will be small for pairs having high sequence identity. However, A-RISC values will be significantly higher than corresponding identities for pairs of proteins with low or medium sequence identities.

## Results

### Papain-like cysteine proteases

In their mature form, the papain-like cysteine proteases (clan CA and family C1) [38] are monomeric proteins composed of approximately 220 amino acids [39–42]. The active site is located between two globular domains (Fig 1B), and the proteolytic activity of these enzymes was reported to contribute to their allergenicity [43–45]. There are several human homologs of these proteins, like cathepsins, that have a similar overall architecture, but have a relatively low sequence identity. For example, human cathepsin K is most similar to Der f 1, as the enzymes share 36% of identical residues [40].

**Fig 2:**
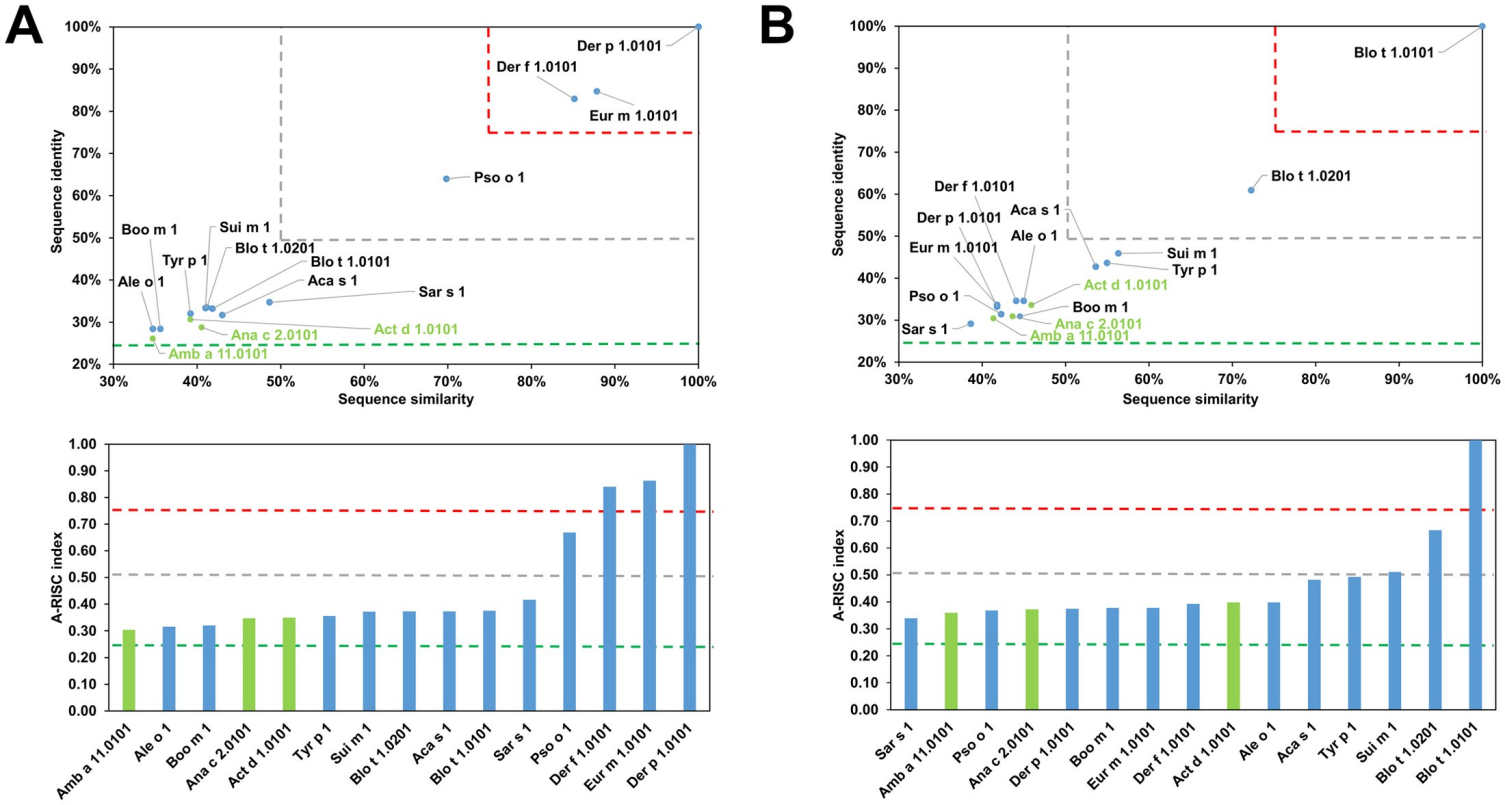
Plots showing relative homology and A-RISC indexes of allergens from the papain-like cysteine protease family. Comparison of other family members to Der p 1.0101 (A) and Blo t 1.0101 (B). Allergens originating from plants are shown in green. Red, gray and dark green dashed lines indicate 75%, 50% and 25% sequence identity and similarity respectively, or 0.75, 0.50 and 0.25 A-RISC values.

This family of allergens contains proteins originating from both animals and plants. Eleven of these proteins originate from mites (Group 1 mite allergens), one from a tick (Boo m 1) and three from plants (Act d 1, Amb a 11 and Ana c 2). The allergens from this family differ significantly in sequence (Table 1). The most frequently recognized allergens from this protein family are excreted by the house dust mites (HDMs), Der p 1 and Der f 1 [46, 47]. Analysis of the relative sequence identities and similarities, as well as the plots with A-RISC index (Fig 2A) clearly show that Der p 1, Der f 1 and Eur m 1 are very similar, and thus, there is a high risk of IgE cross-reactivity between these proteins. Conversely, there is a significantly lower chance of cross-reactivity between the three HDMs allergens and other members of the allergen family. The only exemption is Pso o 1 that has ~64% sequence identity and ~70% similarity to Der p 1, Der f 1 and Eur m 1, and therefore has a significant chance to be involved in a cross-reactive reaction with the aforementioned allergens [48]. In addition, our analysis shows that in case of individuals that were sensitized with Blo t 1.0101 there is a relatively low risk of a cross-reactive reaction with other cysteine proteases presented therein (Fig 2B). Interestingly, in the case of Blo t 1, the difference in sequence between isoallergens (Blo t 1.0101 and Blo t 1.0201) is quite significant. This also clearly highlights differences between proteins originating from HDMs and storage mites and is consistent with limited cross-reactivity between these allergens observed among mite allergic individuals [49–55]. Fig 2B also suggests that a cross-reactive reaction between Blo t 1 is more likely to be observed with Sui m 1 (*Suidasia medanensis*), Tyr p 1 (*Tyrophagus putrescentiae*) and Aca s 1 (*Acarus siro*) than with Der p 1 or Der f 1. This observation is consistent with results of cross-reactivity between allergens from *S*. *medanensis* and *B*. *tropicalis* reported for an urban area of Cartagena (Spain) [56]. In this report, according to RAST and immunoblot inhibition experiments, a significant inhibition of IgE binding to Blo t 1 (corresponding to 25 kDa of *B*. *tropicalis* protein) could be achieved with *S*. *medanensis* extract.

**Fig 3:**
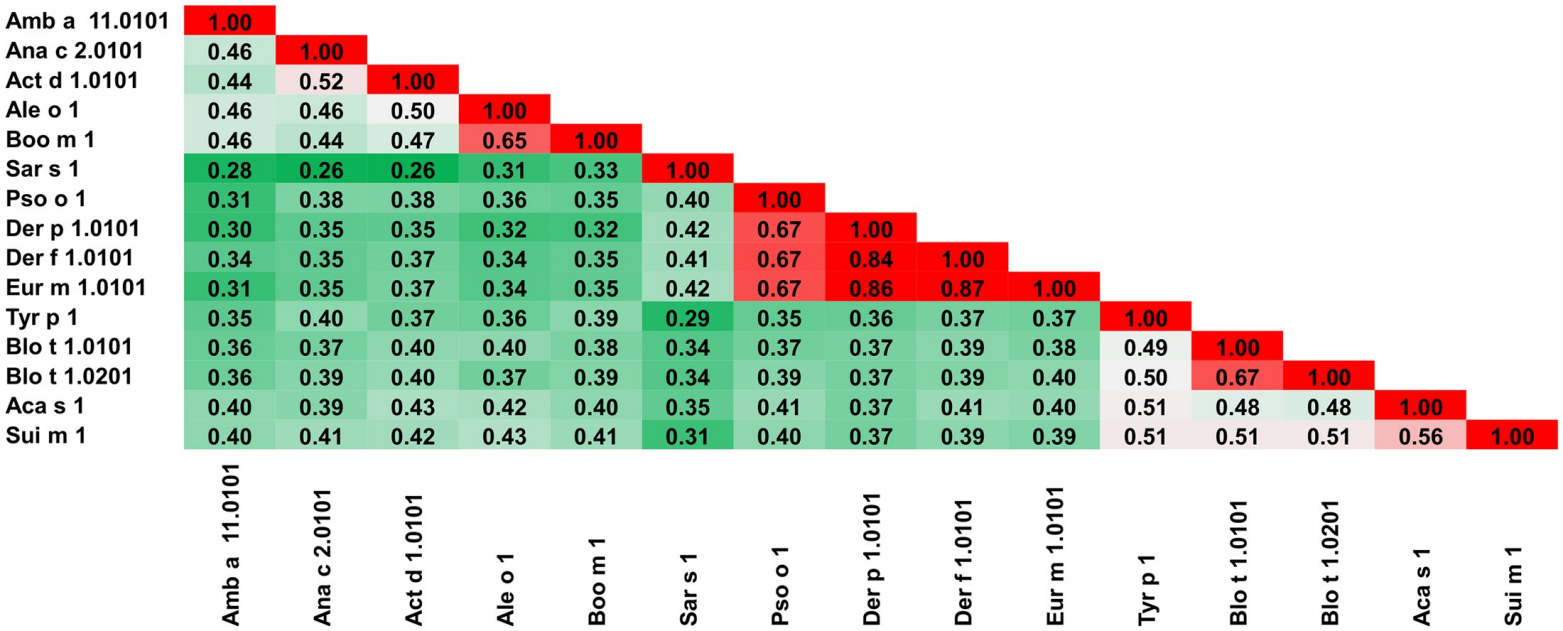
Plot showing A-RISC index values for allergen pairs from the papain-like cysteine protease family. Red color indicates a high risk of cross-reactivity, a dark green color indicates a low risk of cross-reactivity, and intermediate colors correspond to a medium risk of cross-reactivity.

The analysis of A-RISC indexes for the whole family of allergens (Fig 3) shows a low risk of cross-reactivity between allergens originating from mites and plants. The highest risk of cross-reactivity is related to proteins originating from HDMs, whilst that of proteins from storage mites is lower, as they are less similar between themselves. Data presented in Fig 3 also indicate a medium-high risk of cross-reactivity between proteins from *Aleuroglyphus ovatus* (Ale o 1, brown legged grain mite) and *Boophilus microplus* (Boo m 1, southern cattle tick).

### NPC2 family

Proteins belonging to the NPC2 (NPC intracellular cholesterol transporter 2, Niemann-Pick proteins type C2) family are composed of approximately 130 amino acids that form a single β-sandwich domain (immunoglobulin-like tertiary structure) [57–61]. These proteins do not have an enzymatic activity, but they are able to accommodate various ligands [57, 62]. Human NPC intracellular cholesterol transporter 2 shares 25% of identical residues with Der p 2. It was shown that Der p 2 is able to mimic human MD-2, which is a liposaccharide binding component of Toll-like receptor 4 signaling complex, and this molecular mimicry is most likely responsible for Der p 2 allergenicity [63].

**Fig 4:**
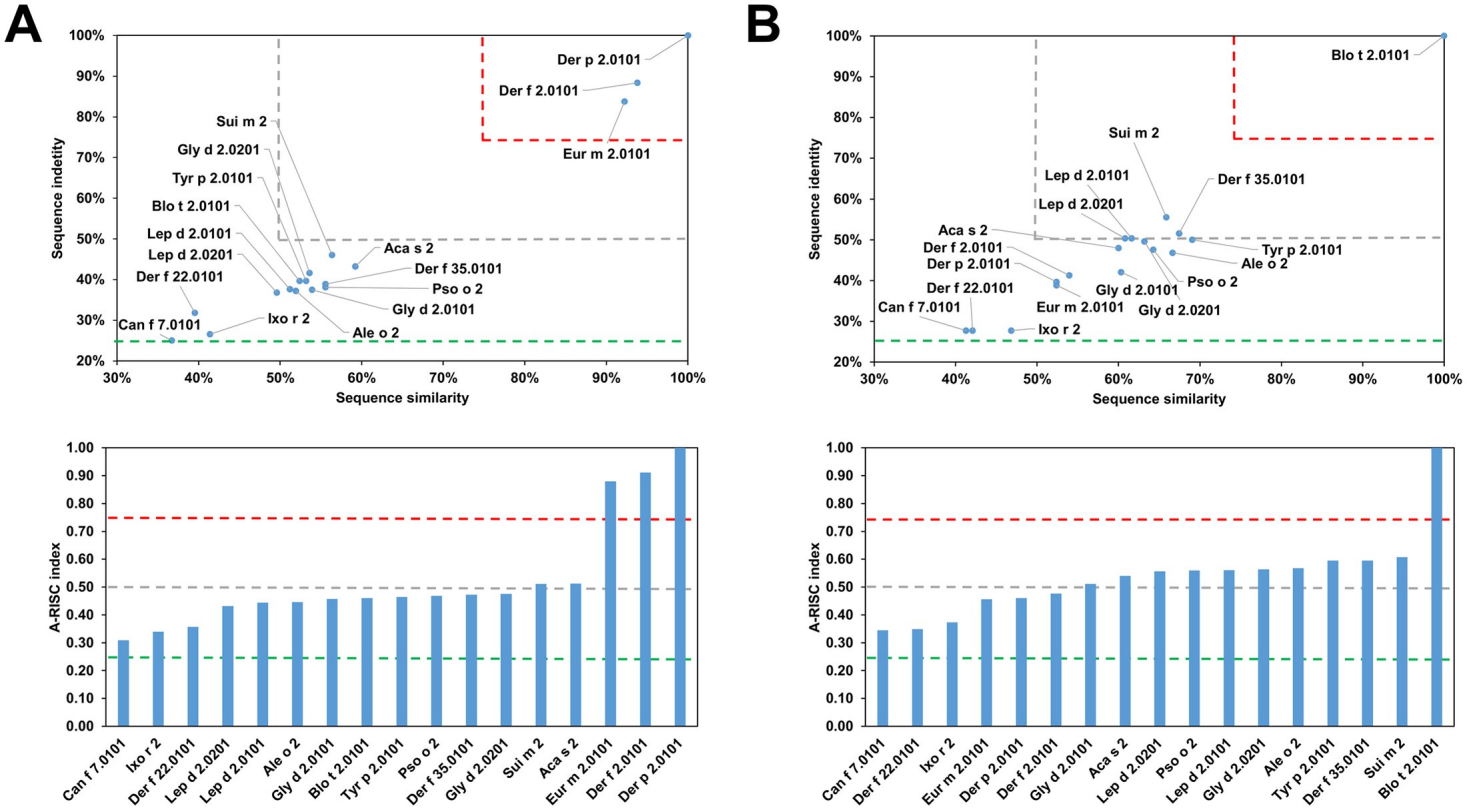
Plots showing relative homology and A-RISC indexes of allergens from the NPC2 family. Comparison of family members to Der p 2.0101 (A) and Blo t 2.0101 (B). Red, gray, and dark green dashed lines indicate respectively 75%, 50% and 25% sequence identity and similarity, or 0.75, 0.50 and 0.25 A-RISC values.

**Fig 5:**
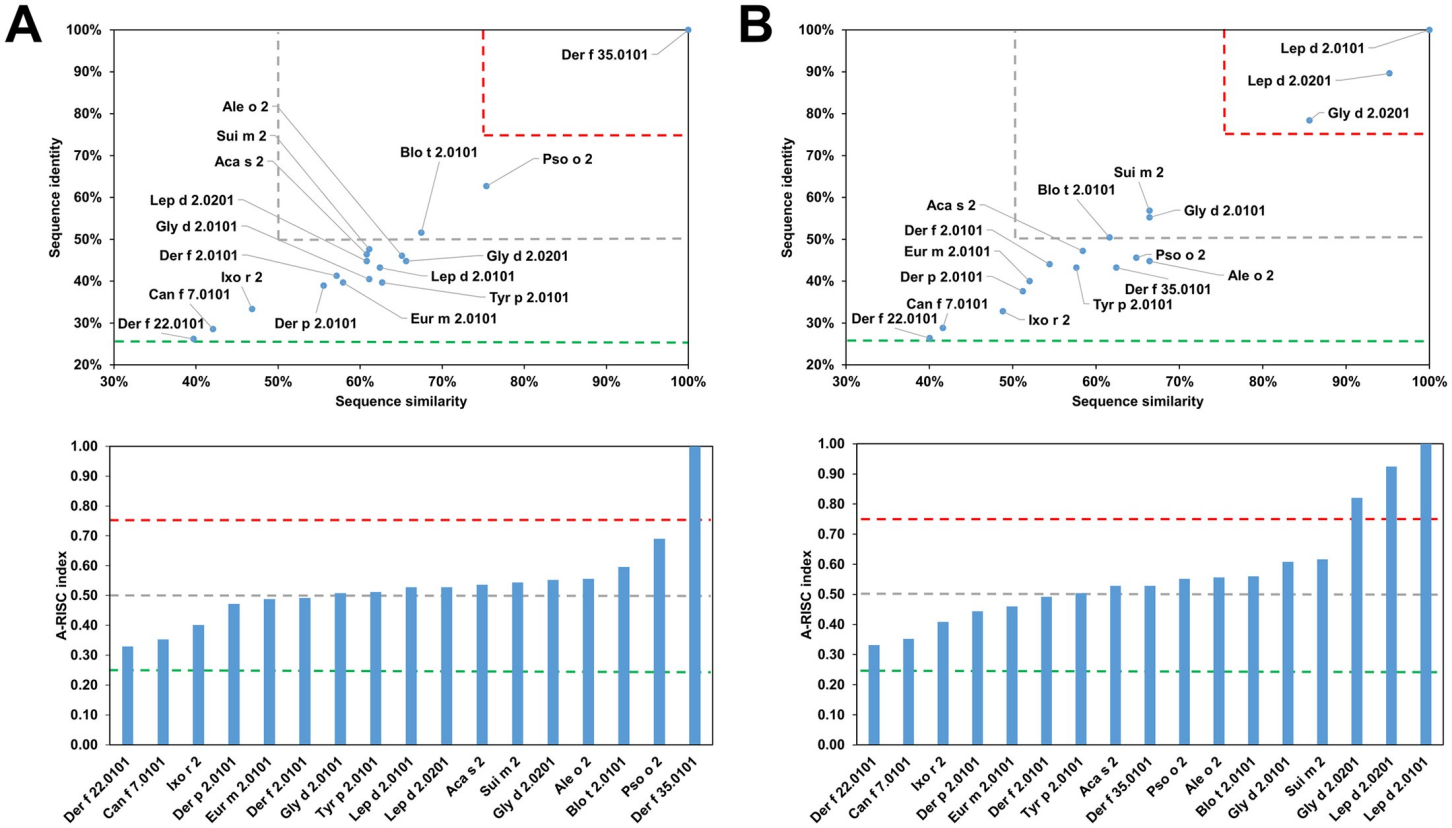
Plots showing relative homology and A-RISC indexes of allergens from the NPC2 family. Comparison of family members to Der f 35.0101 (A) and Lep d 2.0101 (B). Red, gray, and dark green dashed lines indicate respectively 75%, 50% and 25% sequence identity and similarity, or 0.75, 0.50 and 0.25 A-RISC values.

**Fig 6:**
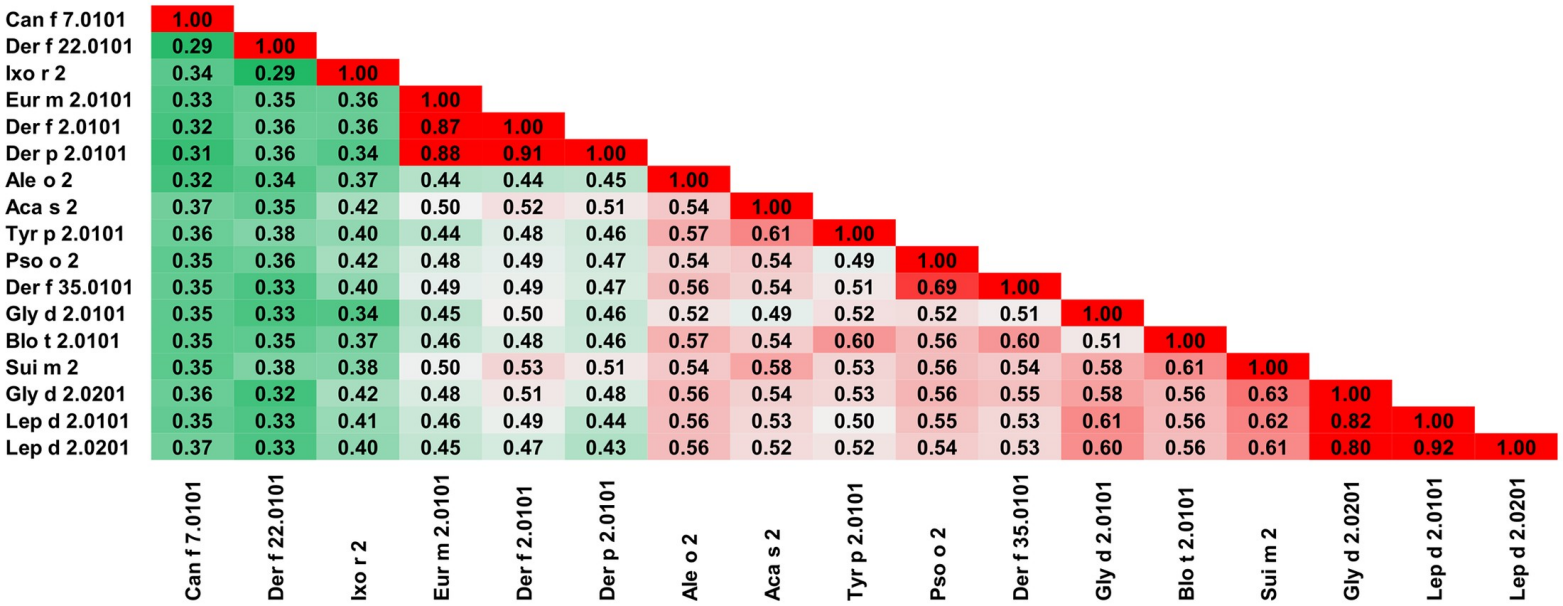
Plot showing A-RISC index values for allergen pairs from the NPC2 family. Red color indicates a high risk of cross-reactivity, a dark green color indicates a low risk of cross-reactivity, and intermediate colors correspond to a medium risk of cross-reactivity.

NPC2 proteins are present in arthropods and vertebrates [64], however allergens from this family originate mainly from mites (Group 2 mite allergens) with only two allergens that have a different source (Can f 7 –dog and Ixo r 2—castor bean tick). Currently, neither food nor plant allergens have been reported as members of this family. Analysis of relative sequence homology shows a situation similar to the case of the cysteine proteases. Namely, Der p 2, Der f 1 and Eur m 2 are similar with sequence identities over 80% and sequence similarities over 90%. Other allergens have less than 50% sequence identity when compared to Der p 2.0101 (Fig 4A). At the same time, Blo t 2 (Fig 4B) is quite distinct from other members of the family with Sui m 2 and Der f 35 having the highest sequence identity of 56% and 52% respectively. Analysis of the sequences of proteins originating from HDMs and storage mites is consistent with observed clinical cross-reactivity. It was shown that there is limited cross-reactivity between Group 2 allergens from *D*. *pteronyssinus* and *B*. *tropicalis*, *Glyciphagus domesticus* or *Lepidoglyphus desctructor*; while there is cross-reactivity between Lep d 2, Gly d 2 and Tyr p 2 [49, 65–67]. These observations are in agreement with our results as depicted on Figs 4 and 5. On the other hand, it was shown that cross-reactivity between Der p 2 and Tyr p 2 may be quite significant [68], despite the sequence identity being below 40%. Furthermore, our analysis reveals that the highest potential risk of cross-reactivity between Group 2 mite allergens originating from HDMs and Blo t 2.0101 is associated with Der f 35.0101, and not Der f 2, Der p 2 or Der f 22 (Fig 6).

There are three allergens belonging to the NPC2 family and originating from *D*. *farinae*: Der f 2, Der f 22 (33%/40% of sequence identity/similarity to Der f 2) and Der f 35 (41%/57% of sequence identity/similarity to Der f 2). Both Der f 2 and Der f 35 are considered to be major allergens, while Der f 22 is a minor allergen [69]. Using these allergens as examples it can be shown that proteins could have quite distinct sequences despite belonging to the same family and originating from a single organism (Figs 4A and 5). On the other hand, the presence of several allergens in one source may cause a complicated pattern of cross-reactive reactions. For example, Der f 35 was shown to be not only cross-reactive with Der f 2, but also with Pso o 2 [69].

Our results also demonstrate that Can f 7 most likely will not be involved in cross-reactive reactions in individuals that are sensitized with Group 2 mite allergens, because this dog allergen shares only 22–31% identical and 37–42% of similar residues with the corresponding mite allergens. A similar situation is observed for Ixo r 2 that shares only 28–34% of identical and 41–50% of similar residues with the corresponding mite allergens.

### Serum albumins

In considering their significant homology to the human serum albumin, serum albumins from other mammals and birds are a very unusual and somewhat unexpected group of allergens [70, 71]. This is because the serum albumins from other mammals analyzed herein have 72–82% sequence identity with the human protein. Serum albumins are also one of the largest allergenic proteins in size; when a single protein chain is considered (~590 amino acids). These allergens have a polypeptide chain that adopts a mostly α-helical conformation and is folded into a three-domain structure. The structure is flexible, which allows serum albumins to bind various small molecular compounds [72]. Sensitization by inhalation to serum albumin alone is quite rare, but these allergens may have a crucial role in cross-reactive reactions in individuals who are sensitized to animal dander [73, 74].

**Fig 7:**
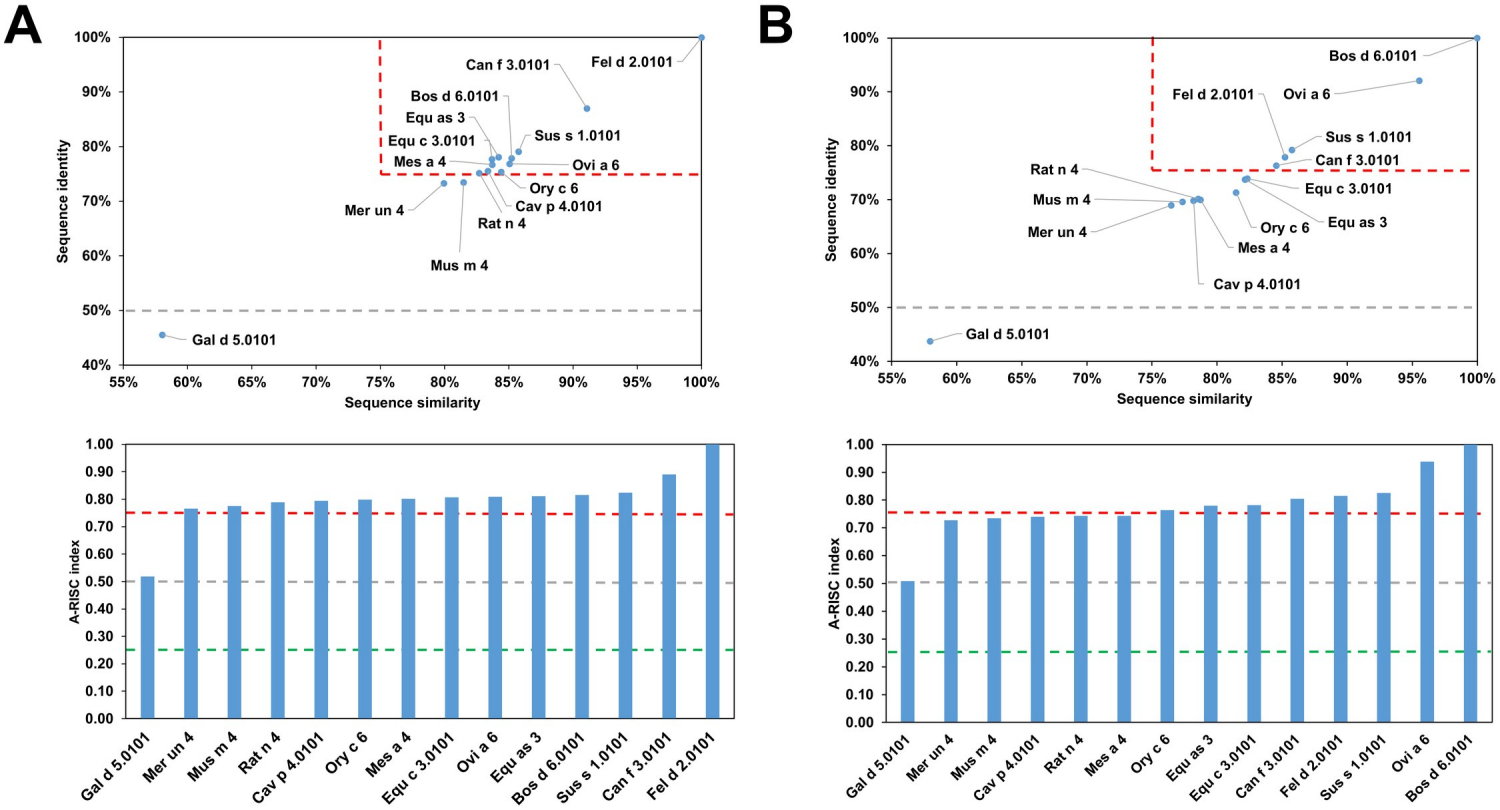
Plots showing relative homology and A-RISC indexes of allergens from serum albumin family. Comparison of family members to Fel d 2.0101 (A) and Bos d 6.0101 (B). Red, gray, and dark green dashed lines indicate respectively 75%, 50% and 25% sequence identity and similarity, or 0.75, 0.50 and 0.25 A-RISC values.

**Fig 8:**
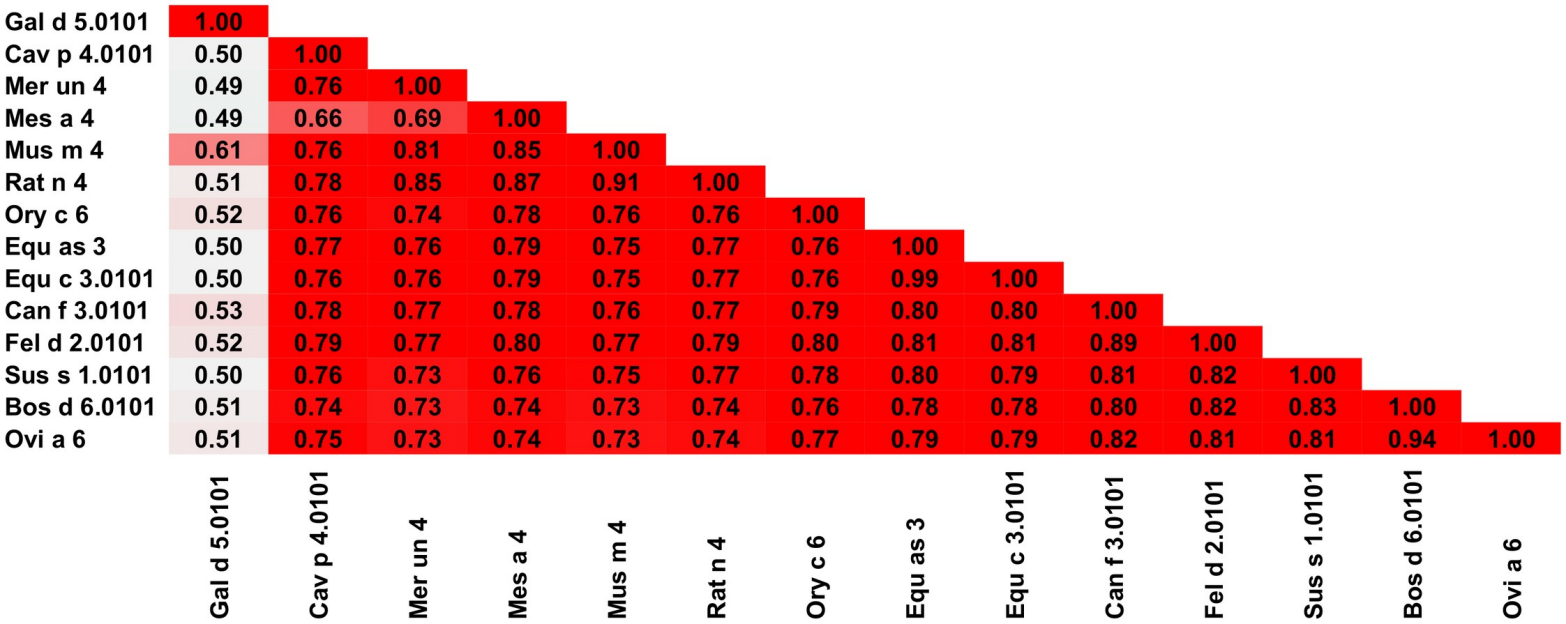
Plot showing A-RISC index values for allergen pairs from the serum albumin family of proteins. Red color indicates a high risk of cross-reactivity, a dark green color indicates a low risk of cross-reactivity, and intermediate colors correspond to a medium risk of cross-reactivity.

Allergens belonging to the serum albumin family of proteins are among the least diverse among protein families that we have analyzed in this manuscript (Table 1, Figs 7 and 8). Thirteen out of fourteen allergenic albumins originate from mammals, Gal d 5 being the only one which originates from birds. The serum albumin family shares a high sequence identity and similarity among its members. Even in the case of Gal d 5, there is a significant level of protein sequence identity (43–46%) and similarity (56–59%) between this protein and other allergens from the family. Such a high level of homology between allergens is reflected by very common cross-reactivity [71, 73–75]. There are multiple examples of cross-reactive reactions that involve serum albumins. For example, the most common cross-reactive reactions involve albumins from cat and pork [76–78], cat and dog [79–81], dog and beef [82], pork and chicken [83], or rodents [73, 75, 84, 85].

Bovine serum albumin is the best studied allergen from this family [86, 87]. Bos d 6 is not only an allergen but is also a protein often used in laboratories and as an additive in the food industry and during preparation of vaccines [88–90]. Although bovine serum albumin is regarded in some cases as a minor allergen, for example in cow dander and serum, it can still be regarded as an important cross-reactive allergen [91]. Our analysis shows that Ova a 6 (albumin from sheep) is the closest in terms of protein sequence to Bos d 6 (92% sequence identity and 96% similarity) and therefore is the most likely candidate to be cross-reactive with the bovine protein. Indeed, this type of cross reactive reaction was reported [92].

### Pectate lyases

**Fig 9:**
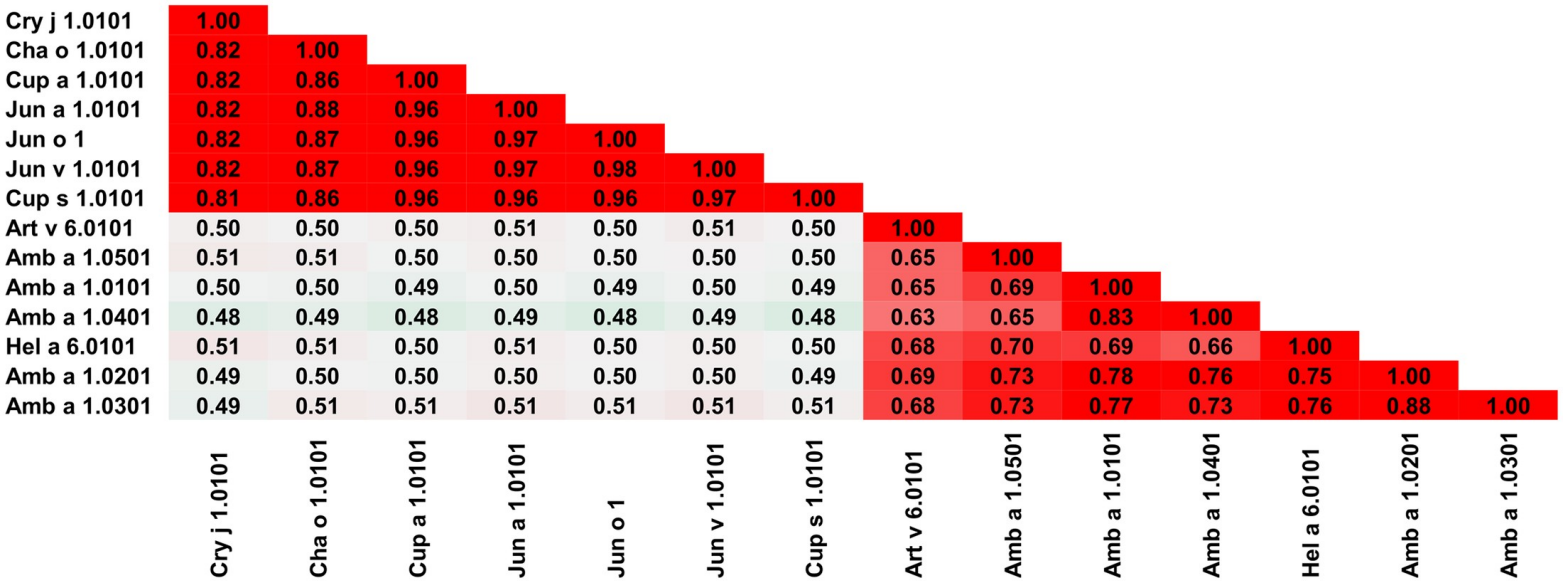
Plot showing A-RISC index values for allergen pairs from the pectate lyase family of proteins. Red color indicates a high risk of cross-reactivity, a dark green color indicates a low risk of cross-reactivity, and intermediate colors correspond to a medium risk of cross-reactivity.

**Fig 10:**
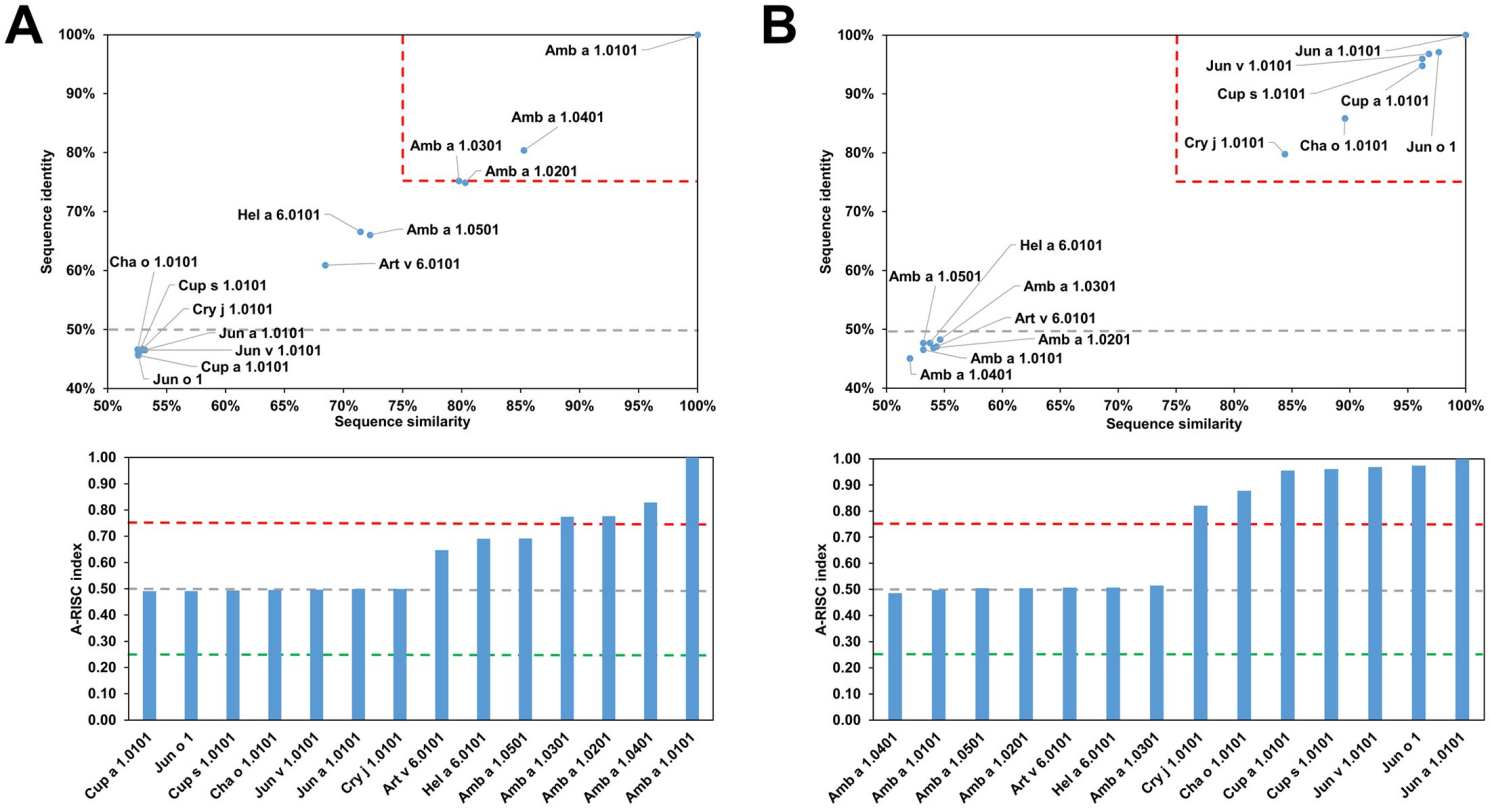
Plots showing relative homology and A-RISC indexes of allergens from pectate lyase family. Comparison of family members to Amb a 1.0101 (A) and Jun a 1.0101 (B). Red, gray, and dark green dashed lines indicate respectively 75%, 50% and 25% sequence identity and similarity, or 0.75, 0.50 and 0.25 A-RISC values.

Pectate lyases are enzymes catalyzing cleavage of pectin which is one of the major cell wall components in higher plants [93]. Pectate lyases originating from plants are relatively large (~340 or ~370 amino acids) and monomeric. The core of the protein is formed by a right-handed β-helix [94]. These proteins are also present in various bacteria and plant pathogens [95]. Currently almost all officially registered allergenic pectate lyases, with the exception of Pen c 32.0101, originate from plants. Pen c 32 is of fungal origin, but it is not included in our analysis, as the complete sequence of this protein is not available [96]. Fig 9 shows that the allergens from this protein family are divided into two distinct groups. The first group includes shorter proteins like Cha o 1, Cry j 1, Cup a 1, Jun a 1, Jun v 1 and Jun o 1, while the second group includes Amb a 1, Art v 6 and Hel a 6 that have an additional N-terminal fragment. The first group of the pectate lyases allergens is less diverse in terms of sequence homology. The second group, despite being more diverse than the first group, contains proteins from fewer sources, and is dominated by various Amb a 1 isoallergens. This phenomenon is also illustrated by Fig 10A, which shows that Amb a 1 isoallergens significantly differ in sequence. Comparison of Amb a 1.0101 or Jun a 1.0101 (Fig 10) with other allergens from this group additionally highlights the presence of the two separate groups of proteins belonging to the pectate lyase family. The two groups emanate from the fact that the allergens from the first group are produced by Cuppressaceae species whilst that of the second group are produced by Asteraceae species [97]. These two species are enlisted amongst the most potent sources of allergens that cause pollen allergies in Europe, North America, and vast parts of Asia. However, even if these two groups of allergens can be separated, there is evidence of cross-reactivity between them [98–101]. It is observed because there is a high degree of homology between the groups and cross-reactivity between them is likely. This conclusion is consistent with observations made by Pichler *et al*. who analyzed sensitization profiles and cross-reactivity patterns between Amb a 1, Art v 6, Cup a 1 and Jun a 1 in individuals from various location of the Northern hemisphere [97]. This study also divided the pectate lyases into four categories (I—Amb a 1, II—Art v 6, III—Cup a 1/Jun a 1 and IV—Cry j 1) according to the ability of the allergens to sensitize predisposed individuals. Such a division is consistent with the location of the mentioned allergens on upper graphs shown in Fig 10.

In some areas of Europe, both ragweed and mugwort are common, and their flowering periods overlap, resulting in some individuals being double-sensitized. In such cases, it is not immediately clear whether the presence of IgE reactivity to both ragweed and mugwort allergens is caused by co-sensitization or cross-reactivity [98]. Either way, both Amb a 1 and Art v 6 may play important roles and be primary sensitizers [98, 99]. However, it was also suggested that, at least in the areas where patients are exposed to both ragweed and mugwort pollen, Amb a 1 has more IgE epitopes than Art v 6, hence, it may elicit more diverse IgE and T cell responses than Art v 6, as well as dominate the cross-reactivity with the mugwort homolog [99]. However, in regions where there is high mugwort pollen exposure, Art v 6 can be the primary sensitizing agent; and inevitably by epitope cross-recognition of T and B cells facilitate sensitization to Amb a 1.

Amb a 1 is the major and the most studied ragweed allergen with several characterized isoallergens (Fig 10A). In fact, a detailed analysis of a purified sample from ragweed pollen Amb a 1 isoallergens (01, 02 and 03) revealed that Amb a 1.01 had the highest IgE-binding reactivity. At the same time, both Amb a 1.01 and Amb a 1.03 were potent stimulators of human T cells [102]. Unfortunately, these studies did not include Amb a 1.04 or Amb a 1.05 and therefore it is not possible to compare experimentally derived IgE-binding or T cell stimulatory properties of all Amb a 1 isoallergens. Another study showed that all five Amb a 1 isoallergens are present in pollen with Amb a 1.01 and Amb a 1.03 being the most often recognized [103].

### Lipocalins

Lipocalins, approximately 200 residues in length, are proteins that show great diversity at the sequence level (Table 1) and yet, they share the same overall structure with an eight-stranded central β-barrel [104–106]. These proteins illustrate excellently the limits of sequence similarity when used for identification of structures with a similar fold; and that preservation of the proteins’ overall folds in many cases is more important than the preservation of a particular sequence. In fact, it was shown that less than 10% of protein pairs with sequence identity below 25% have similar structures, and that proteins are rarely homologous when the level of sequence identity drops below 10% [107]. Some lipocalins emphasize these findings.

The oligomeric state of lipocalins further complicates deciphering their biological structures, because they can be either monomeric or oligomeric; and it was also shown that the quaternary structure may depend on protein concentration [108]. Among inhalant allergens, lipocalins are regarded as one of the most important group of animal allergens [109]. Glycosylation of some of these lipocalin proteins that are involved in allergic reactions makes allergy diagnosis more challenging [109]. Although lipocalins have a relatively poor capacity to stimulate immune cells, they are able to induce very high IgE production in atopic individuals [110]. Moreover, lipocalins are versatile ligand carriers [109, 111–113] and are involved in various biological processes. For example, there are 37 lipocalins identified in the human genome alone. These lipocalins are important for human physiological processes as they are believed to play specific roles in some diseases [114].

**Fig 11:**
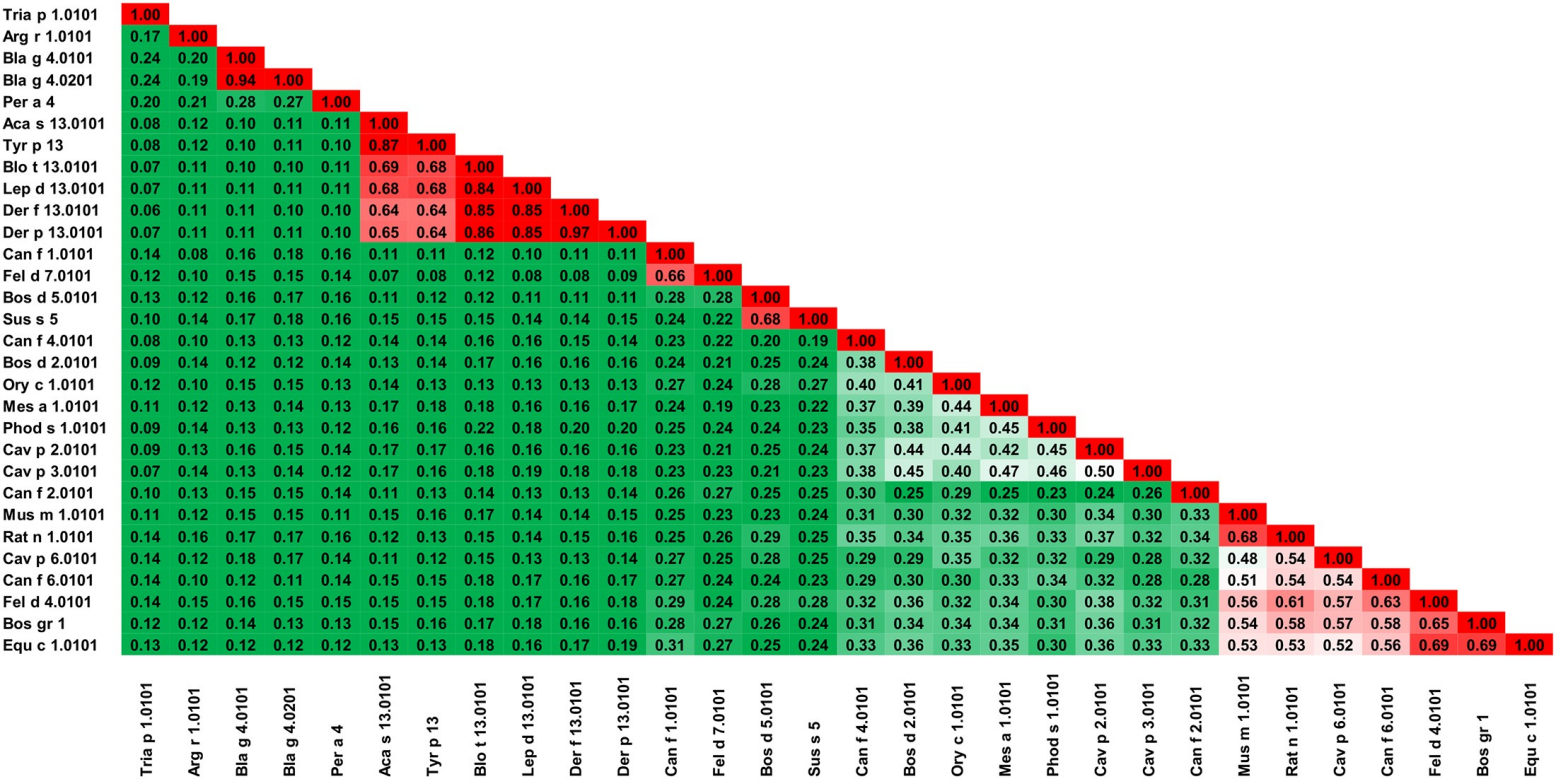
Plot showing A-RISC index values for allergen pairs from lipocalin family of proteins. Red color indicates a high risk of cross-reactivity, a dark green color indicates a low risk of cross-reactivity, and intermediate colors correspond to a medium risk of cross-reactivity.

**Fig 12:**
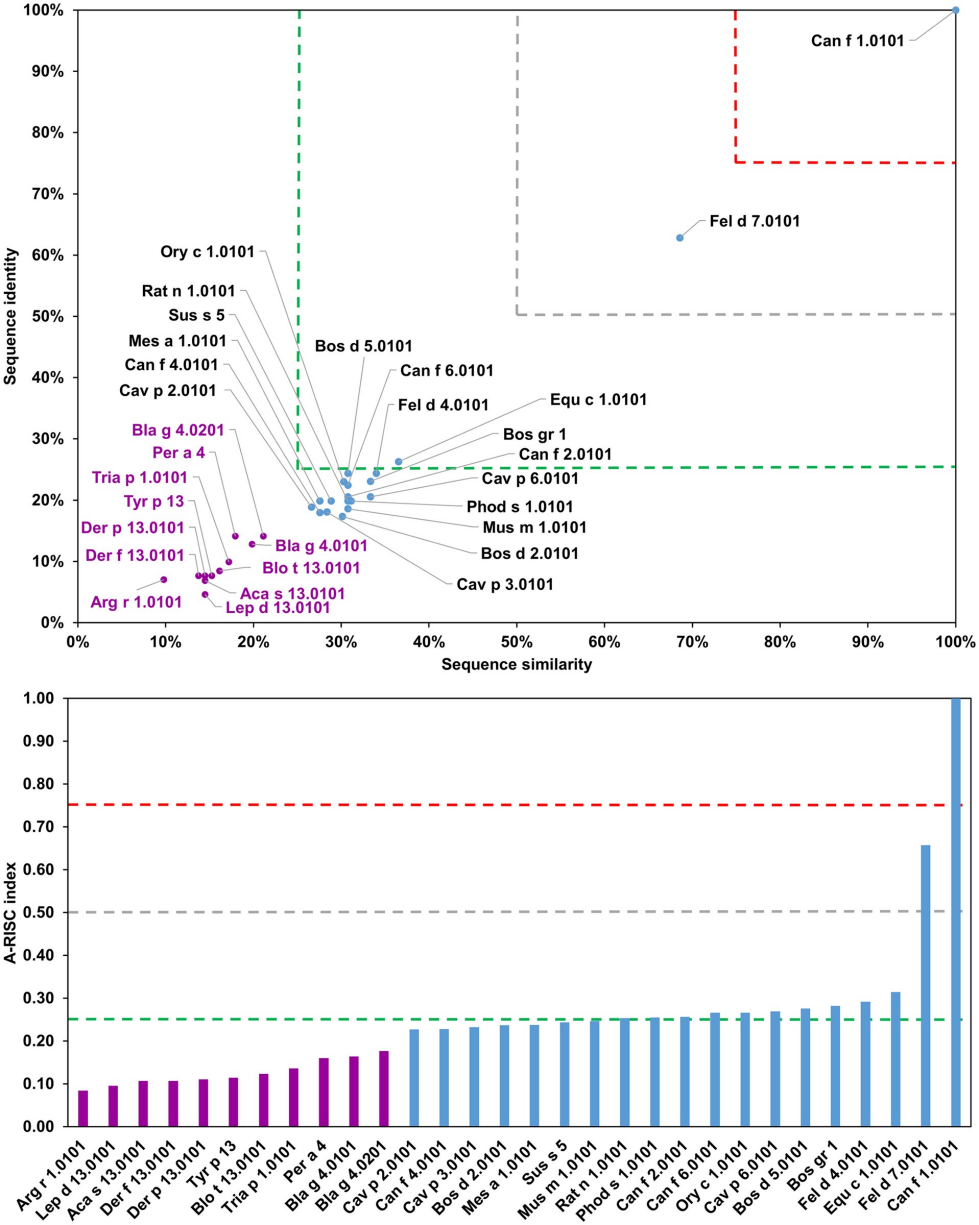
Plots showing relative homology and A-RISC indexes of allergens from lipocalin family of proteins. Comparison of family members to Can f 1.0101. Allergens originating from Arthropods are marked in purple, while mammalian lipocalins are shown in blue. Red, gray and dark green dashed lines indicate respectively 75%, 50% and 25% sequence identity and similarity, or 0.75, 0.50 and 0.25 A-RISC values.

**Fig 13:**
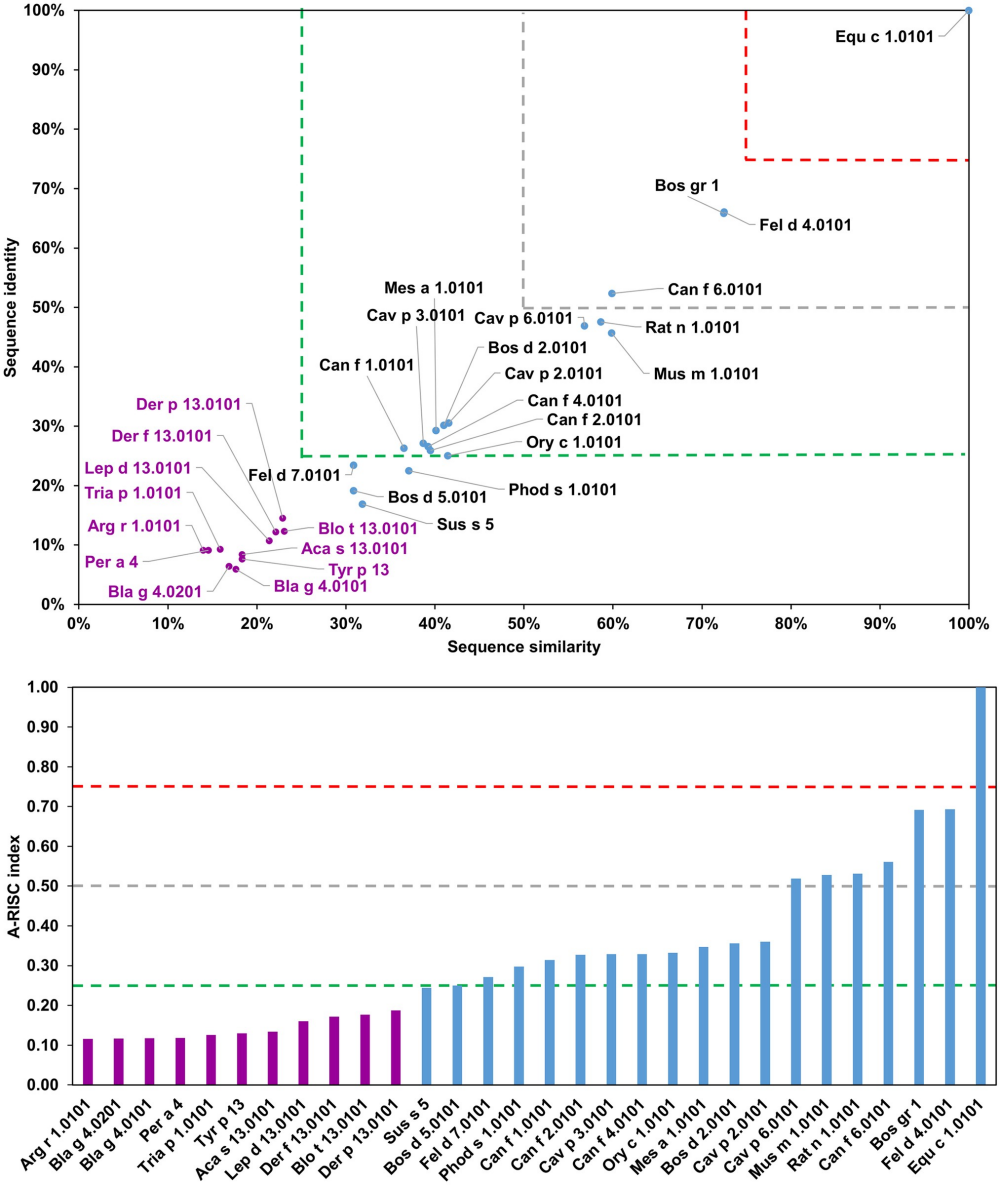
Plots showing relative homology and A-RISC indexes of allergens from lipocalin family of proteins. Comparison of family members to Equ c 1.0101. Allergens originating from Arthropods are marked in purple, while mammalian lipocalins are shown in blue. Red, gray, and dark green dashed lines indicate respectively 75%, 50% and 25% sequence identity and similarity, or 0.75, 0.50 and 0.25 A-RISC values.

The sequence diversity and a relatively large number of representative allergens from this protein family (Fig 11), makes their analysis especially interesting. Our analysis allows us to divide these allergens into seven subgroups. The first subgroup includes proteins from insects (Bla g 4, Per a 4, Tria p 1) and ticks (Arg r 1), which have low probability of cross-reactive reactions between them. The second subgroup contains lipocalins from mites (Aca s 13, Blo t 13, Der f 13, Der p 13, Lep d 13 and Tyr p 13), and according to our results (Fig 11), there is a high risk of cross-reactivity between these proteins. The third subgroup consists of Can f 1 and Fel d 7 (63% sequence identity), and the fourth group includes Bos d 5 and Sus s 5 (64% sequence identity) (Fig 12). Bos d 2, Can f 4, Cav p 2, Cav p 3, Mes a 1, Ory c 1 and Phod s 1 comprise the fifth subgroup. These proteins have 29–43% sequence identity (A-RISC indexes 0.35–0.50), and we classify them as associated with a medium-low risk of cross-reactivity. The sixth subgroup has only one representative—Can f 2 that is most similar to Rat n 1 with 28% sequence identity. Subgroup seven includes Bos gr 1, Can f 6, Cav p 6, Equ c 1, Fel d 4, Mus m 1 and Rat n 1 (Fig 13). These lipocalins share 43–66% of identical amino acids and may be classified as having medium-high likelihood of being involved in cross-reactive reactions.

Allergens from dog (Can f 1, Can f 2, Can f 4 and Can f 6) are the largest group of lipocalin allergens originating from a single source. These molecules share only 17–24% of identical residues, but, cross-reactivity was reported between Can f 1 and Can f 2 despite the dissimilarities [115, 116]. The same studies also showed cross-reactivity between Can f 1 and human tear lipocalin [115]. Can f 1 is also cross-reactive with Fel d 7 which is not surprising when considering the high sequence identity between the proteins (Fig 12) [117]. Cross-reactivity between Can f 4 and Bos d 2 was reported as well [118]. This agrees with our classification of allergenic lipocalins, as both these allergens belong to subgroup five that we proposed. Clinically relevant cross-reactivity between Can f 6, Equ c 1, and Fel d 4 was reported [119, 120] and can be explained by a high homology of these allergens (they belong to subgroup seven that we suggested; Fig 11).

### β-Expansins and expansin-related proteins

**Fig 14:**
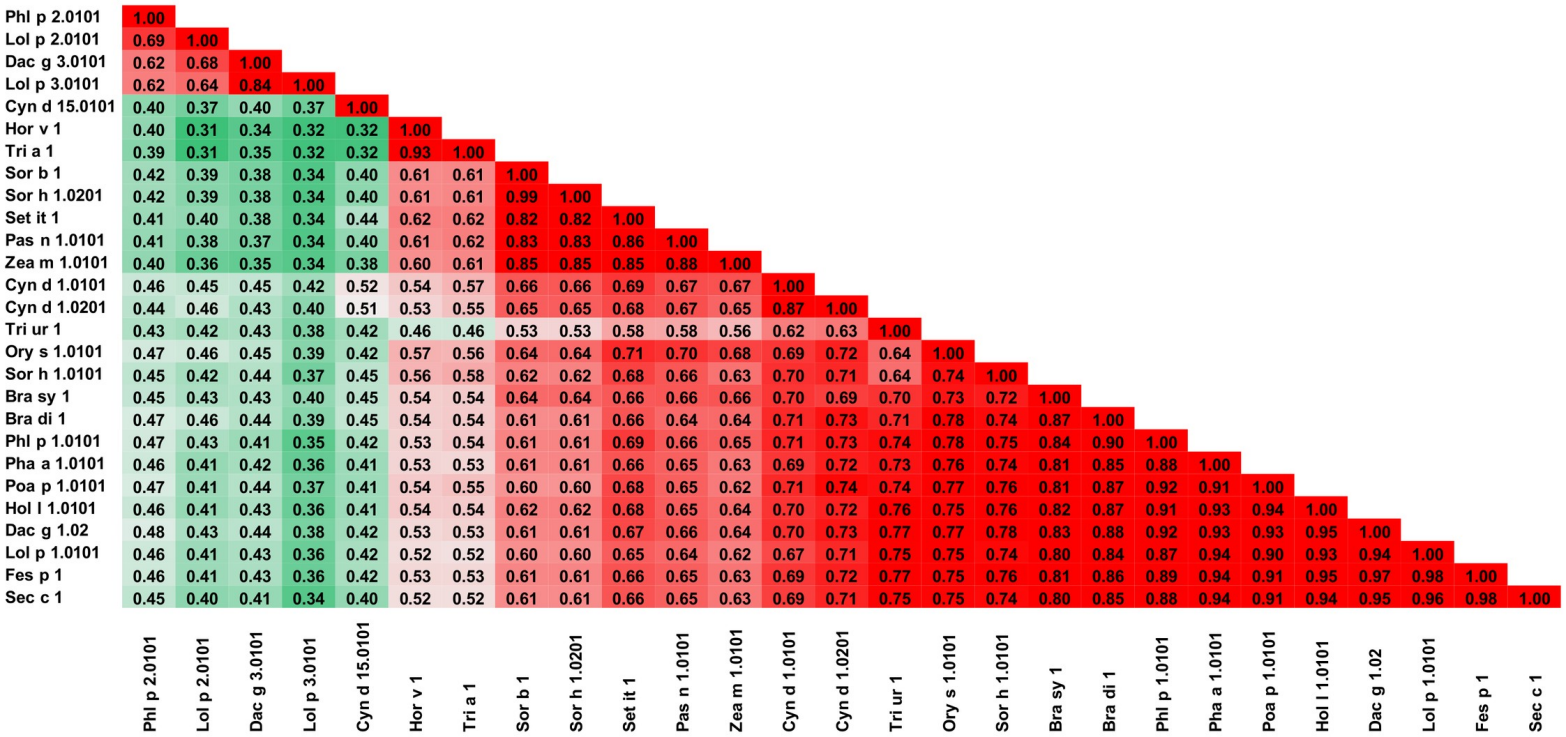
Plot showing A-RISC index values for allergen pairs from the β-expansin family and expansin-related proteins. Red color indicates a high risk of cross-reactivity, a dark green color indicates a low risk of cross-reactivity, and intermediate colors correspond to a medium risk of cross-reactivity.

The expansin superfamily includes four protein families: α-expansin, β-expansin, expansin-like A and expansin-like B [121–124]. The expansin superfamily originates from plants and is believed to take part in plant developmental processes when cell wall modification and cell expansion occurs [124]. Expansin proteins are most often composed of 250–275 amino acids that are folded into molecules with two domains [124]. The first (N-terminal) domain is similar to a catalytic domain of the glycoside hydrolase family 45 of proteins, and the second (C-terminal) domain is similar to Group-II pollen allergens. Group I pollen allergens belong to the β-expansin family of proteins, while Group II pollen allergens most likely evolved from a truncated β-expansin ancestor gene [124]. Therefore, Group II allergens contain only domain 2 (95–100 amino acids) which is characteristic of expansins. From a protein classification point of view, Group II pollen allergens do not belong to expansin superfamily; they form a separate family of proteins [124]. However, from the allergy perspective it is important to analyze these two protein families together, as they are still similar in terms of sequence and domain structure. The division between Groups I and II is visualized in Fig 14.

**Fig 15:**
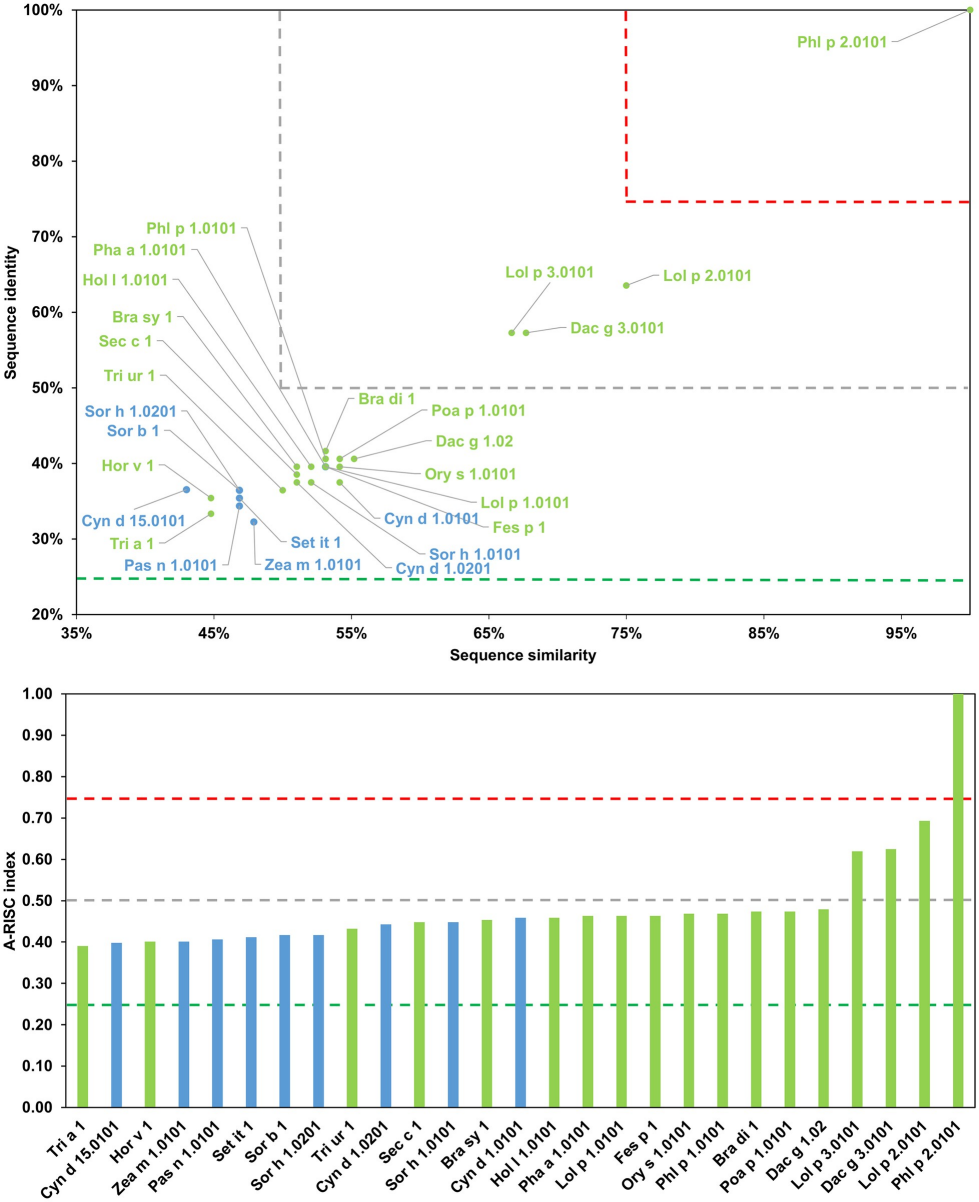
Plots showing relative homology and A-RISC indexes of allergens from β-expansin family of proteins. Comparison of family members to Phl p 2.0101. Temperate grasses are marked in green, while subtropical grasses are marked in blue. Red, gray and dark green dashed lines indicate respectively 75%, 50% and 25% sequence identity and similarity, or 0.75, 0.50 and 0.25 A-RISC values.

**Fig 16:**
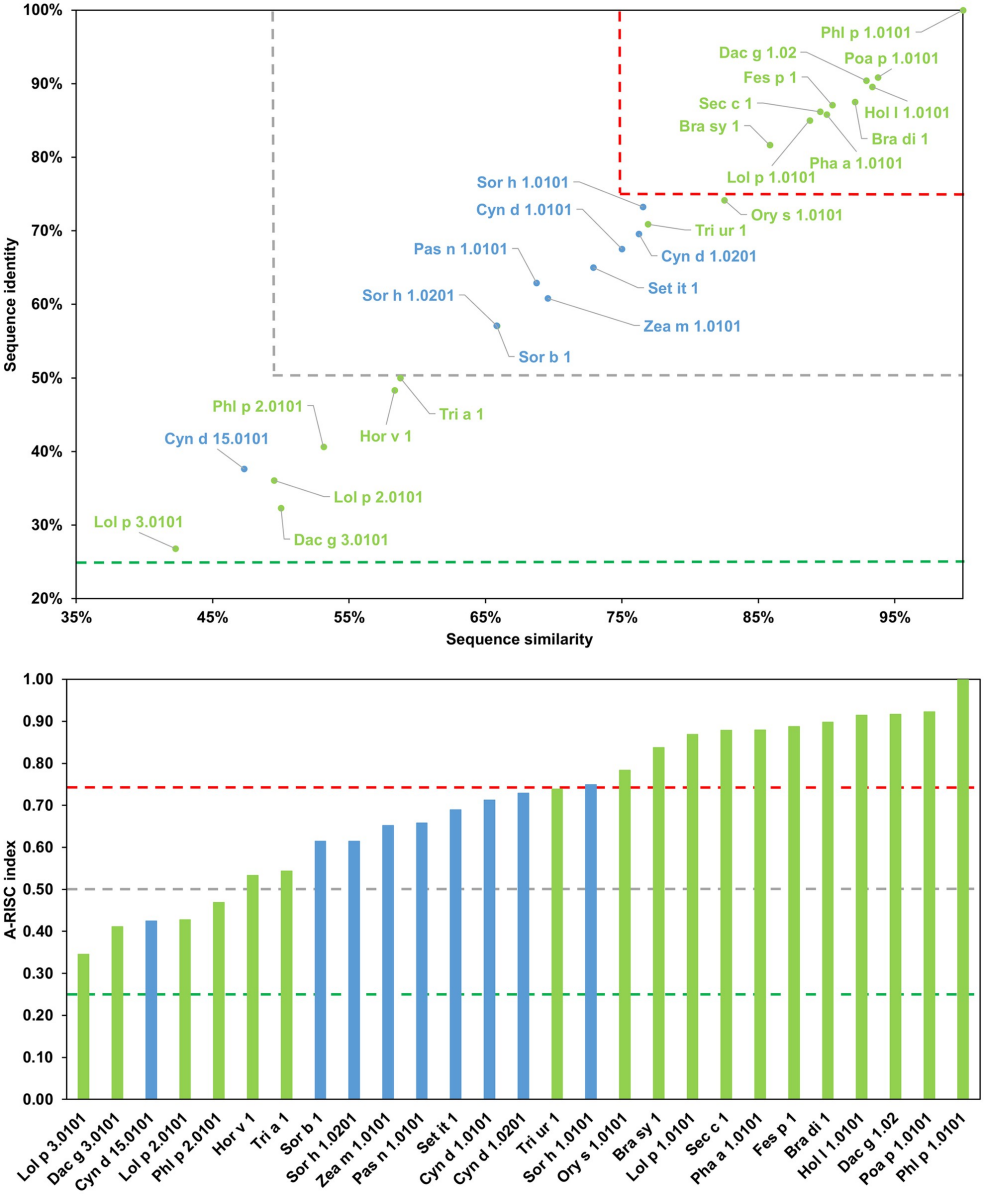
Plots showing relative homology and A-RISC indexes of allergens from β-expansin family of proteins. Comparison of family members to Phl p 1.0101. Temperate grasses are marked in green, while subtropical grasses are marked in blue. Red, gray and dark green dashed lines indicate respectively 75%, 50% and 25% sequence identity and similarity, or 0.75, 0.50 and 0.25 A-RISC values.

The analysis of this group of proteins allows us to divide the allergens into the following subgroups: I—Dac g 3, Lol p 2, Lol p 3 and Phl p 2 (Fig 15), II—Cyn d 15, III—Hor v 1 and Tri a 1, IV—Pas n 1, Sor b 1, Sor h 1.02, Set it 1 and Zea m 1, V–Bra di 1, Bra sy 1, Cyn d 1, Dac g 1, Fes p 1, Hol l 1, Lol p 1, Ory s 1, Pha a 1, Phl p 1, Poa p 1, Sec c 1, Sor h 1.01 and Tri ur 1 (Fig 16). A likelihood of cross-reactivity between allergens from subgroup I may be estimated as medium-high or high. Nonetheless, these allergens are less likely to be cross-reactive with proteins from other subgroups. Interestingly though, such a cross-reactivity is observed, as most of the IgE-binding epitopes of Hol l 1, Lol p 1 and Phl p 1 are located in the C-terminal domain 2 of these β-expansins, and this domain is present in Group II/III allergens [125, 126]. This confirms the probability of cross-reactivity between these subgroups. It is also worth mentioning that multiple IgE antibodies can bind to Phl p 1, some of which recognize the N-terminal part of the protein [126].

On a different note, Cyn d 15 contains only domain 2 and is therefore classified as a β-expansin-related protein. Even though it is similar in size to allergens from subgroup I, Cyn d 15 has a distinct sequence (Fig 15), which is related to the fact that it originates from a subtropical grass (Bermuda grass; Chloridoideae subfamily), while all members of the subgroup I originate from grasses growing in temperate zones (Pooideae subfamily, Fig 14) [127, 128]. Subgroup III members (Hor v1 and Tri a 1) are close homologs (91% sequence identity, A-RISC index- 0.93), and an estimated risk of the cross-reactivity with other proteins from Groups I-III of pollen allergens is medium. Subgroup IV (Pas n 1, Sor b 1, Sor h 1.02, Set it 1 and Zea m 1) includes β-expansins from subtropical grasses and there is a high risk of cross-reactivity between members because these allergens share 82–99% of identical residues.

Concurrently, the likelihood of cross-reactivity between members of subgroup IV and subgroups III and V may be estimated as medium-high (Fig 14). Subgroup V is the most numerous but can be divided into two separate subgroups: Va (first subgroup) with Cyn d 1.01, Cyn d 1.02, Ory s 1, Sor h 1.01 and Tri ur 1, and Vb (second subgroup) with Bra di 1, Bra sy 1, Dac g 1, Fes p 1, Hol l 1, Lol p 1, Pha a 1, Phl p 1, Poa p 1 and Sec c 1. Such a division separates subtropical and temperate zones grasses [127]. Interestingly, although the likelihood of cross-reactivity between members of the subgroup Va is relatively high (A-RISC indexes 0.62–0.78), it is lower than the cross-reactivity between members of the subgroup Vb (A-RISC indexes 0.80–0.98). This observation is consistent with experimental data that shows an extensive cross-reactivity between Pooideae grasses [128, 129] and is also visible in Fig 16 when comparing Phl p 1 to other members of this allergen group. In summary, the likelihood of cross-reactivity between β-expansins from subgroups III-V is medium-high to high according the A-RISC classification, which agrees with experimental data [130–133].

### Group V/VI grass allergens

The allergen family including Group V/VI grass pollen allergens has significantly less members in comparison with the β-expansins and expansin-related allergens family. Most likely, this is caused by the fact that this family of grass allergens contains only proteins originating from Pooideae (temperate grasses) and no representative proteins from subtropical grasses [127, 128, 134]. Group V/VI grass pollen allergens are responsible for causing Type I allergy in atopic individuals which can be up to 20% of the population in North America and Europe [135]. These proteins are composed of 240–310 amino acids that fold into two α-helical domains connected by a flexible linker [136]. The only exception is Phl p 6.0101, with 110 amino acids in the protein chain [137, 138]. This is due to the fact that Phl p 6.0101 has only residues corresponding to the N-terminal domain of the Group V grass pollen allergens. The modular architecture of the Group V grass pollen allergens and the presence of the flexible linker region allows for a relatively easy fragmentation of these proteins [139].

**Fig 17:**
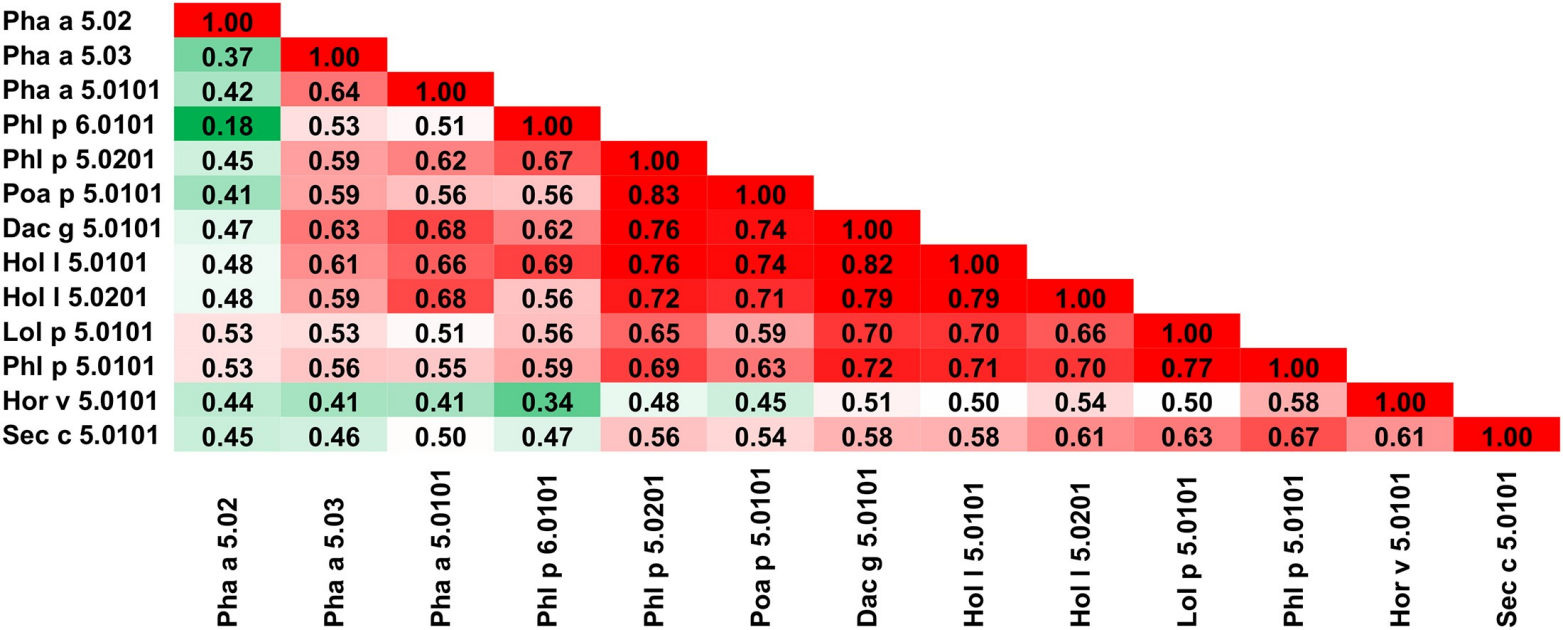
Plot showing A-RISC index values for allergen pairs from Group V/VI grass pollen allergens. Red color indicates a high risk of cross-reactivity, a dark green color indicates a low risk of cross-reactivity, and intermediate colors correspond to a medium risk of cross-reactivity.

**Fig 18:**
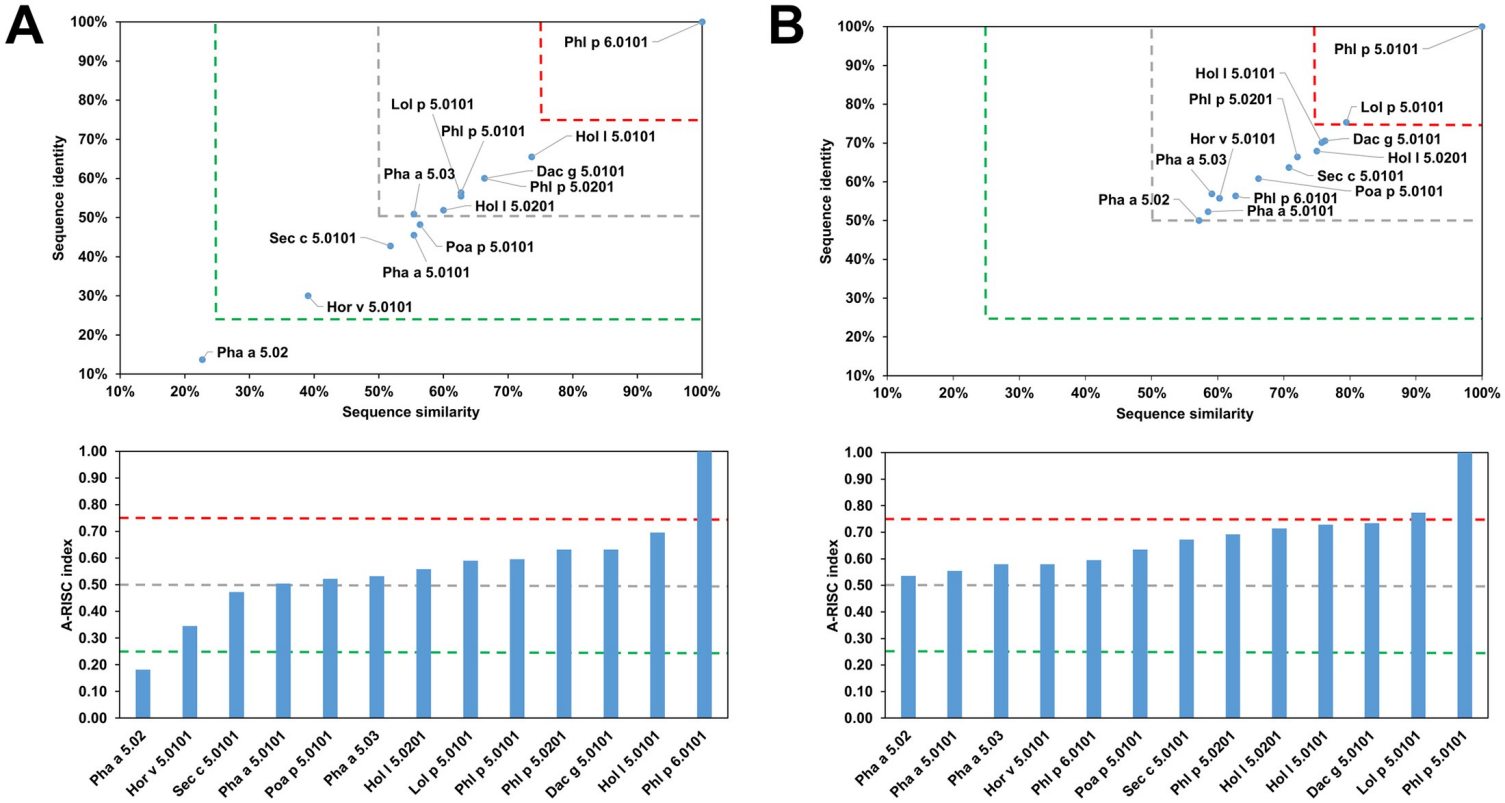
Plots showing relative homology and A-RISC indexes of allergens from Group V/VI grass pollen family. Comparison of family members to Phl p 6.0101 (A) and Phl p 5.0101 (B). Red, gray and dark green dashed lines indicate respectively 75%, 50% and 25% sequence identity and similarity, or 0.75, 0.50 and 0.25 A-RISC values.

It is more difficult to divide this family into subgroups (Fig 17), as there is no clear pattern describing similarities between family members. Pha a 5.02 is quite distinct from other members of the family (Figs 17 and 18). Phl p 6.0101 is most similar to Hol v 5.0101 (65% sequence identity, A-RISC index 0.70) and least similar to Pha a 5.02 (14% sequence identity; Fig 18A). Phl p 6.0101 may be grouped together with Pha a 5.03 and Pha a 5.0101, as these three have a relatively low similarity to Pha a 5.02, Hor v 5.0101 and Sec c 5.0101; however, they are quite similar to the remaining members of the family. The largest subgroup delineated includes Dac g 5.0101, Hol l 5.0101, Hol l 5.0201, Lol p 5.0101, Phl p 5.0101, Phl p 5.0201 and Poa 5.0101, and these allergens are most similar among the members of the family and therefore there is a medium-high or high likelihood of a cross-reactivity between them (Fig 17), which is consistent with observed patterns of clinical cross-reactivity [128, 132, 140–142]. Phl p 5.0101 (Fig 18B) and Lol p 5.0101 are also distinct as they both are similar to all members of the family (Figs 17 and 18B) [143–147].

### Profilins

Profilins were identified as allergens almost four decades ago [148, 149]. They are small (~130 amino acids), multifunctional proteins present in either a monomeric or dimeric form [150]. One of the most important feature of these molecules is their ability to interact with many proteins by binding to the poly-proline fragments [151–158]. The most important function of profilins in cells is related to the interaction with actin [159, 160]. This interaction plays a crucial role in various cellular processes like endocytosis, membrane trafficking, regulation of neuronal plasticity and/or cell motility.

**Fig 19:**
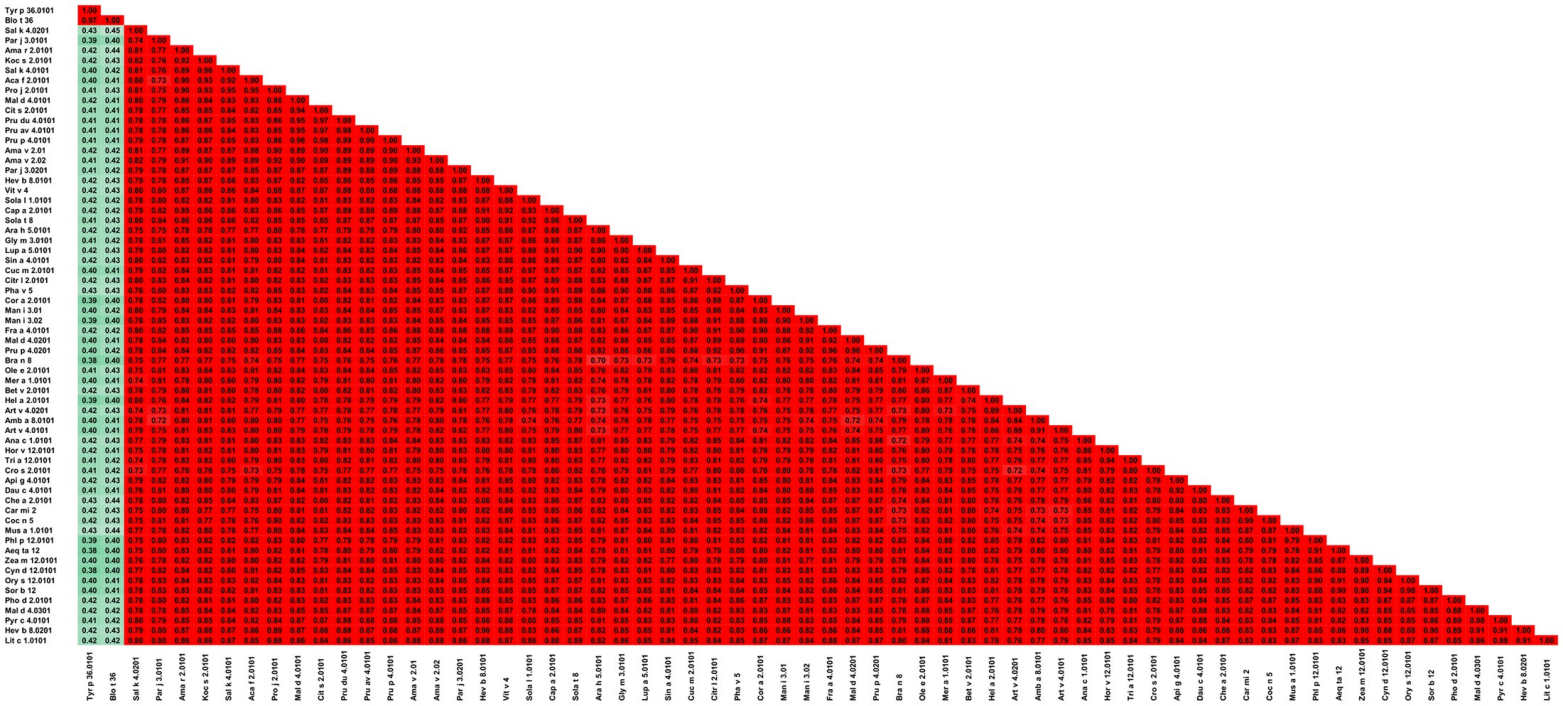
Plot showing A-RISC index values for allergen pairs from profilin family of proteins. Red color indicates a high risk of cross-reactivity, a dark green color indicates a low risk of cross-reactivity, and intermediate colors correspond to a medium risk of cross-reactivity.

**Fig 20:**
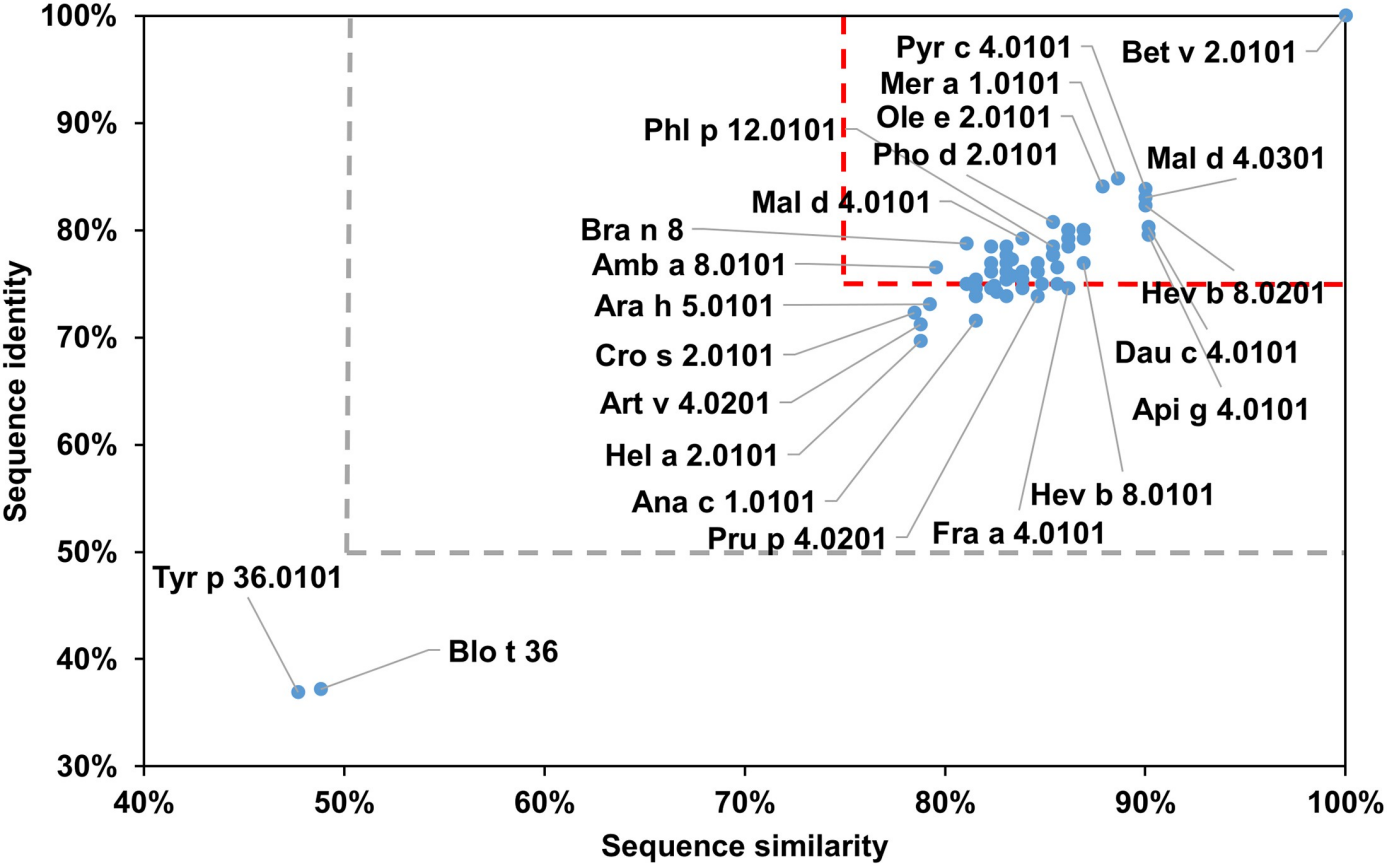
Plots showing a relative homology of allergens from profilin family. Comparison of family members to Bet v 2.0101. Only selected members of the family are labeled. Red and gray dashed lines indicate respectively 75% and 50% sequence identity and similarity, or 0.75, and 0.50 A-RISC values.

Profilins are highly conserved in terms of their sequence (Table 1, Fig 19) and structure as they are considered as the only true panallergens [161]. Profilins originating from plants are especially similar and they are responsible for various cross-reactive reactions [162, 163]. This is consistent with our results as A-RISC index values for plant profilins are in 0.73–99 range. In this respect the profilin family is very similar to serum albumin family. The two profilins originating from storage mites (Blo t 36 and Tyr p 36) are very similar to each other, but they significantly differ in sequence when compared to plant profilins (Figs 19 and 20). Allergens from this family are structurally similar to human profilins, however sequence identity with human homologs is smaller than 25% [164–166].

The most common reactions involving allergens from the profilin family are associated with Oral Allergy Syndrome (OAS; also known as Pollen Food Syndrome) which is considered a class II food allergy. In this syndrome, an allergen from pollen is the sensitizer while the related food allergen is the elicitor of symptoms [167]. The origin of OAS may be explained using Fig 20 as an illustration. In this case, it is assumed that birch pollen profilin (Bet v 2.0101) is responsible for the sensitization, and due to the high homology with food profilins, for example from apple (Mal d 4.0301), pear (Pyr c 4.0101), carrot (Dau c 4.0101), and celery (Api g 4.0101), cross-reactive reactions are observed. This is also reflected by clinical sensitization patterns. In Northern Europe, where sensitization to birch allergen is common, Bet v 1 and Bet v 2 are responsible for birch-celery syndrome. A similar situation is observed in the case of OAS involving birch and apple, where both PR-10 and profilin allergens are involved in cross-reactive reactions [168]. In the areas where there is no exposure to birch allergens, profilins from weeds (Amb a 8 and Art v 4), grass (Phl p 2), olive (Ole e 2) or date palm (Pho d 2) may replace Bet v 2 as potential sensitizing allergens [169–171]. This results in various pollen-food syndromes including mugwort-celery, mugwort-peach, ragweed-melon-banana, grass-fruit, plane-fruit, etc. [172–179]. In summary, the comparison of profilins sequences not only shows why cross-reactivity between these allergens is so wide-spread, but also explains the high-risk of such events. It also shows why using one profilin may be sufficient in diagnosing sensitization to plant profilins [180–182].

### Non-specific lipid transport proteins

**Fig 21:**
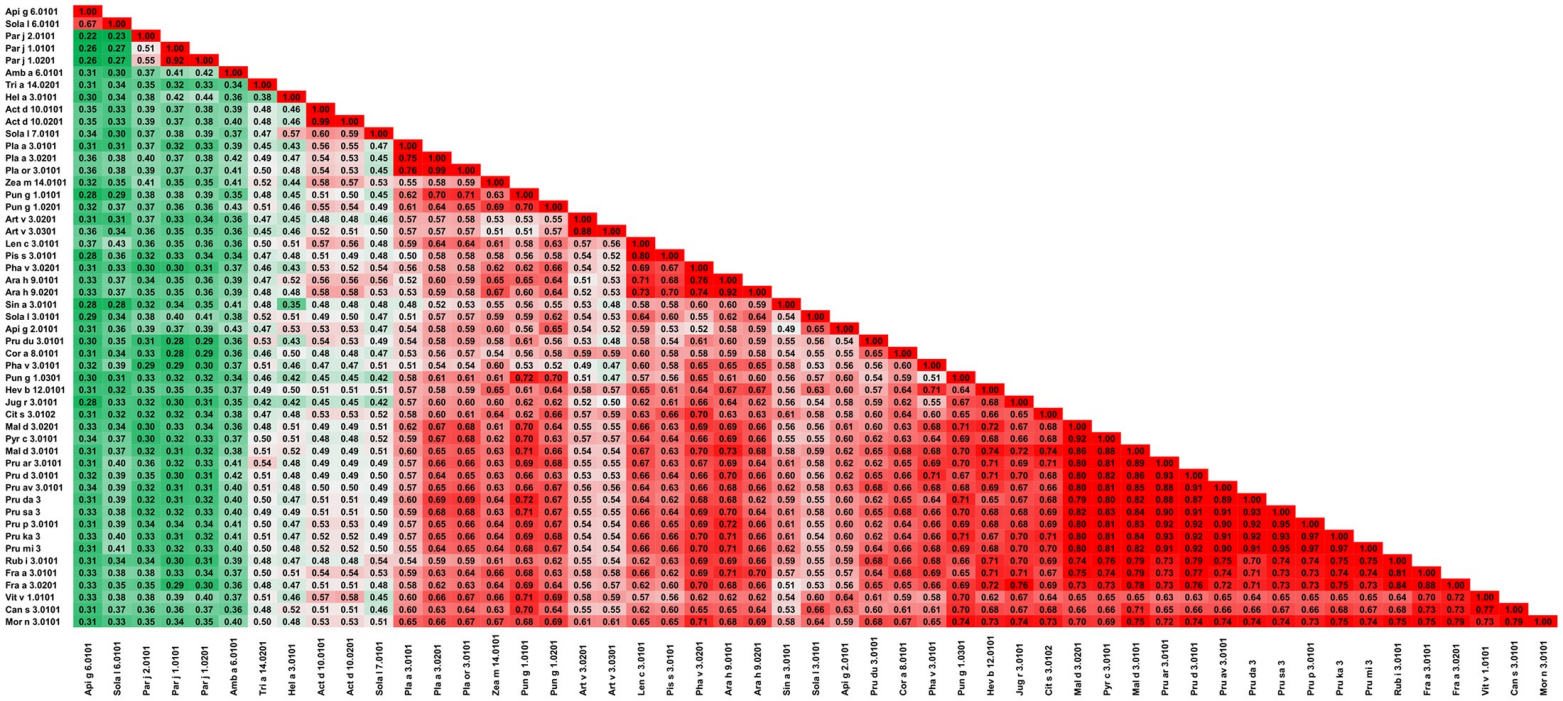
Plot showing A-RISC index values for allergen pairs from nsLTP family. Red color indicates a high risk of cross-reactivity, a dark green color indicates a low risk of cross-reactivity, and intermediate colors correspond to a medium risk of cross-reactivity.

Non-specific lipid transport proteins (nsLTPs) form one of the largest families of allergens and they are classified as panallergens [183]. These plant derived molecules are very diverse in terms of their primary structure (Fig 21), and include both food and inhaled allergens. NsLTPs are considered to be the most frequent food allergens in Mediterranean countries [184]. NsLTPs together with cereal prolamins, 2S-albumins, bifunctional α-amylase/protease inhibitors, soybean hydrophobic protein, indolines, and α-globulins belong to the prolamin superfamily of proteins [4, 5, 185–188]. This family can be divided further into nsLTP1 and nsLTP2, from which the first category includes proteins composed of ~90 amino acids, whereas the second category contains even smaller proteins (~70 amino acids) [189–191]. NsLTPs are folded into a bundle of four or five α-helices stabilized by four conserved disulfide bridges. This type of overall structure provides nsLTPs with unusual stability that allows some of these allergens to survive thermal processing and digestion [192–195]. It was shown that, following thermal denaturation, Cor a 8 refolds in acidic conditions similar to ones observed in the human stomach [196]. The α-helices of a nsLTP form a hydrophobic cavity that can accommodate various ligands [189, 190]. Interestingly, in the case of Pru du 3, a bound ligand makes the protein more susceptible to gastroduodenal proteolysis [197].

It is also worth noting that a particular source of allergens may contain several of nsLTPs that are on the official list of the World Health Organization and International Union of Immunological Societies (WHO/IUIS) Allergen Nomenclature Sub-committee. For example, there are three allergens originating from tomato (Sola l 3, Sola 6 and Sola l 7) and three from peanut (Ara h 9, Ara h 16 and Ara h 17). In addition, there are many isoallergens identified for this family of proteins. Based on Fig 21, it is possible to divide allergenic nsLTPs into several subgroups. The first subgroup includes Amb a 6.0101, Api g 6.0101, Sola l 6.0101, Par j 10101, Par j 1.0201 and Par j 2.0101. These proteins are quite distinct from the remaining allergens, and because of this, there is a relatively low probability of a cross-reactive reaction between this subgroup and other subgroups. However, within the subgroup I, there is a relatively high risk of cross-reactivity between Api g 6.0101 and Sola l 6.0101, as well as between nsLTPs from *P*. *judaica*. This predicted cross reactivity is consistent with clinical observations, as the IgE cross-reactivity between Par j 1 and Par j 2 was demonstrated [198]. The second subgroup includes Act d 10.0101, Act d 10.0201, Hel a 3.0101, Sola l 7.0101, and Tria a 14.0201. Analysis of this subgroup suggests that Sola l 7.0101 is likely the greatest culprit of the reported nsLTP-related tomato-sunflower cross-reactivity [199]. In addition, it highlights a medium-high probability of cross-reactivity between kiwi fruit (Act d 10) and grape (Vit v 1.0101), maize (Zea m 14.0101), peanut (Ara h 9.0201), and/or tomato (Sola l 7.0101). The third subgroup is composed of Pla a 3.0101, Pla a 3.0201, Pla or 3.0101, Pun 1.0101, Pun g 1.0201, and Zea m 14.0101. These proteins are not only similar to each other, but at the same time, they have significant homology to other nsLTPs, excluding most allergens from subgroups I and II. This is consistent with the observed cross-reactivity between Pla a 3 or Pan g 1with Pru p 3 [200–202].

**Fig 22:**
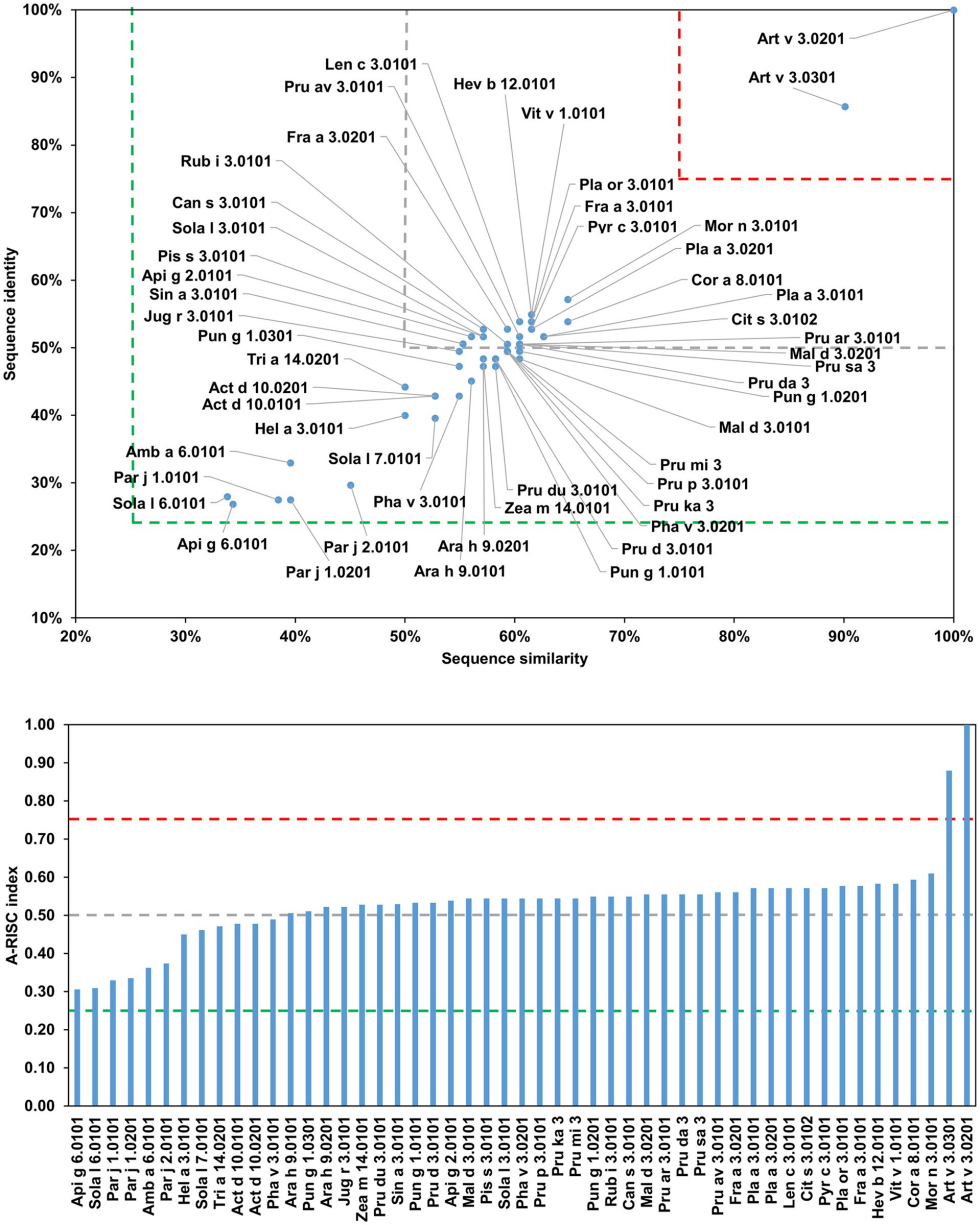
Plots showing relative homology and A-RISC indexes of allergens from nsLTP family. Comparison of the family members to Art v 3.0201. Red, gray and dark green dashed lines indicate respectively 75%, 50% and 25% sequence identity and similarity, or 0.75, 0.50 and 0.25 A-RISC values.

The fourth subgroup contains only Art v 3.0201 and Art v 3.0301 (Fig 22), as these two proteins are similar to each other, but distinct from remaining members of the family. Art v 3.0101 is not considered, as there is no complete sequence available for this allergen. Comparison of Art v 3 with other members of the family (Fig 22) suggests a medium-high risk of cross-reactivity with most of these allergens. This is also observed in clinical studies that showed cross-reactivity between Art v 3 and Pru p 3 or Ara h 9 [203–206]. Lombardero *et al*. also reported lack of cross-reactivity between Art v 3 and Par j 1 [203].

**Fig 23:**
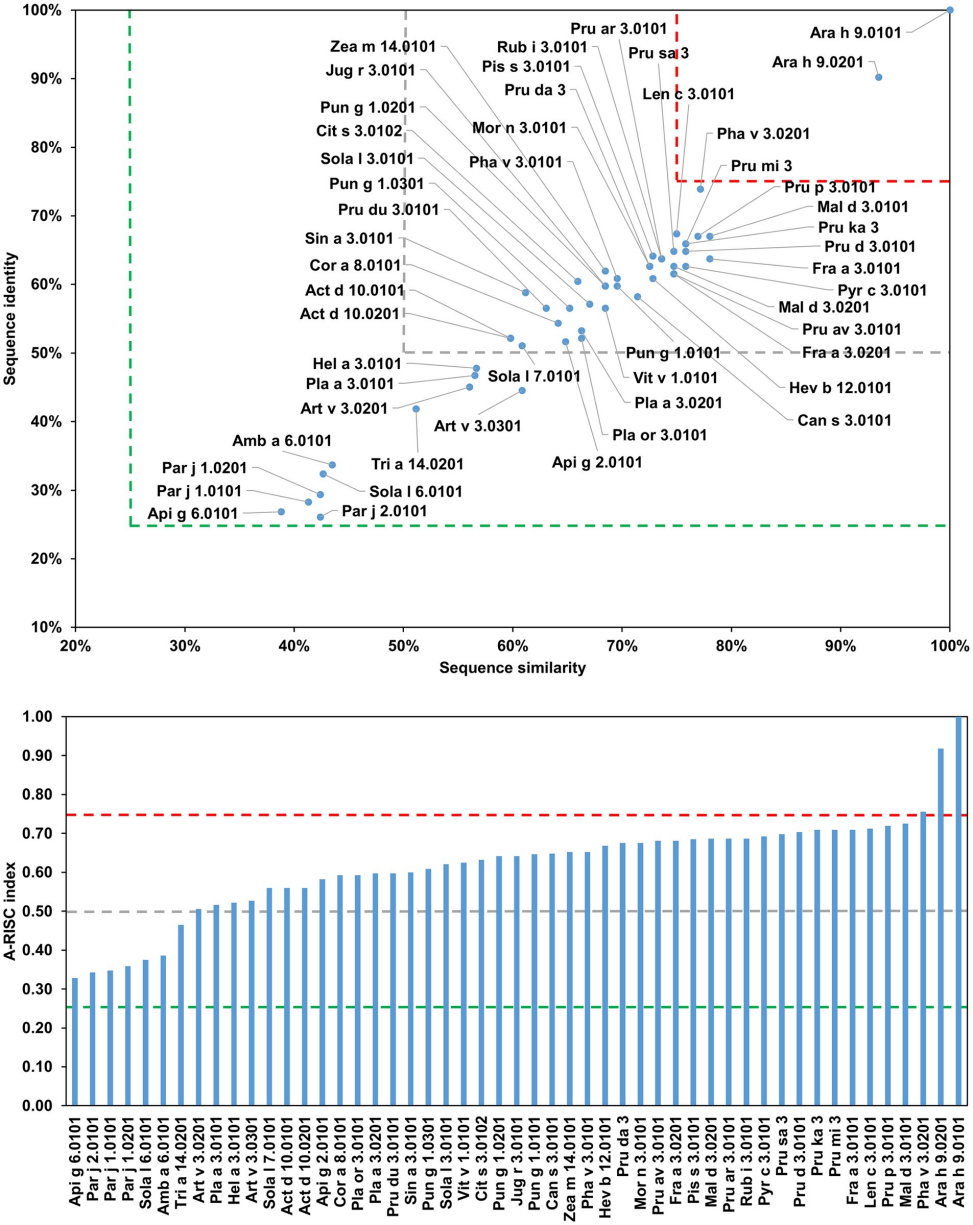
Plots showing relative homology and A-RISC indexes of allergens from nsLTP family. Comparison of the family members to Ara h 9.0101. Red, gray, and dark green dashed lines indicate respectively 75%, 50% and 25% sequence identity and similarity, or 0.75, 0.50 and 0.25 A-RISC values.

The fifth subgroup includes proteins from the Fabales order: Ara h 9.0101, Ara h 9.0201, Len c 3.0101, Pha v 3.0201 and Pis s 3.0101. The A-RISC values (Fig 23) suggest a medium-low risk of cross-reactivity between these proteins and members of subgroup I, and a medium-high likelihood of cross-reactivity with other members of the family. The risk of cross-reactivity within the subgroup is relatively high.

It is also worth noting that the analysis also predicts a high risk of a cross-reactivity between Ara h 9.0101 and Mal d 3.0101, Pru p 3.0101 or Fra a 3.0101 (Figs 21 and 23). The sixth group includes Api g 2.0101, Cit s 3.0102, Can s 3.0101, Cor a 8.0101, Hev b 12.0101, Jug r 3.0101, Mor n 3.0101, Pha v 3.0101, Pru du 3.0101, Pun g 1.0301, Sin a 3.0101, Sola l 3.0101 and Vit v 1.0101. Within the subgroup, a likelihood of cross-reactivity is estimated to be mostly medium-high.

**Fig 24:**
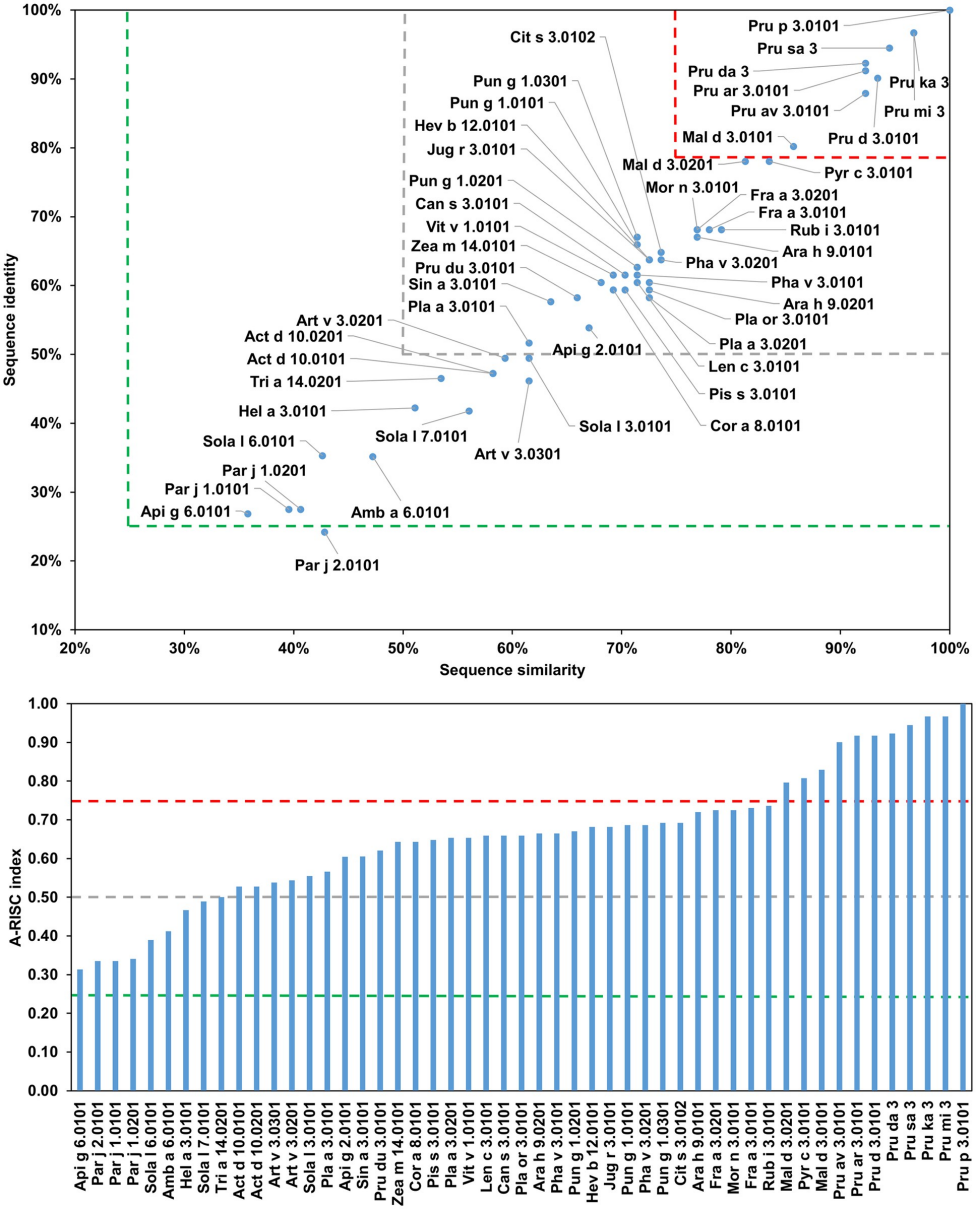
Plots showing relative homology and A-RISC indexes of allergens from nsLTP family. Comparison of the family members to Pru p 3.0101. Red, gray and dark green dashed lines indicate respectively 75%, 50% and 25% sequence identity and similarity, or 0.75, 0.50 and 0.25 A-RISC values.

The last subgroup (VII), which is also the most numerous, includes Fra a 3.0101, Fra a 3.0201, Mal d 3.0101, Mal d 3.0201, Pru ar 3.0101, Pru av 3.0101, Pru d 3.0101, Pru da 3, Pru ka 3, Pru mi 3, Pru p 3.0101, Pru sa 3, Pyr c 3.0101, and Rub i 3.0101. These allergens are similar to each other and comprise a group of allergenic nsLTPs for which there is the highest risk of cross-reactivity (Fig 21). This subgroup also contains Pru p 3 that it is believed to be found in the Mediterranean area and is supposedly the main culprit responsible for nsLTP sensitization [207–211]. Pru p 3 is also the most extensively studied allergen from this family. Fig 24 clearly illustrates why the peach nsLTP may be easily involved in cross-reactive reactions with homologous proteins originating for example from apple, apricot, cherry, plum or raspberry.

### PR-10 (Bet v 1—Like) family

The PR-10 allergens constitute one of the largest group, with 26 allergens currently registered on the official list of allergens published by the International Union of Immunological Societies (IUIS) website. However, this number increases over 100 when all PR-10-related isoallergens registered by IUIS are considered. It is important to stress that while Bet v 1-like allergens are produced by many genes with varying expression patterns [212–214], the resulting proteins have almost no post-translational modifications [215–217]. However, simultaneously, the natural mixture of these allergens can be very complex and may contain many isoallergens, only some of which are clinically relevant [218]. Nevertheless, PR-10 proteins are an important group of panallergens, and they are present in many plants. Proteins from this family are composed of approximately 160 amino acids that adopt a characteristic fold of a seven stranded anti-parallel β-sheet and three α-helices [219–221]. A PR-10 molecule contains a large cavity that is capable of binding various ligands [222–224]. Furthermore, using Bet v 1, it was shown that the binding of ligands is isoform dependent, and it was also suggested that the ligands may play a role in enhancement of allergic sensitization [224, 225].

**Fig 25:**
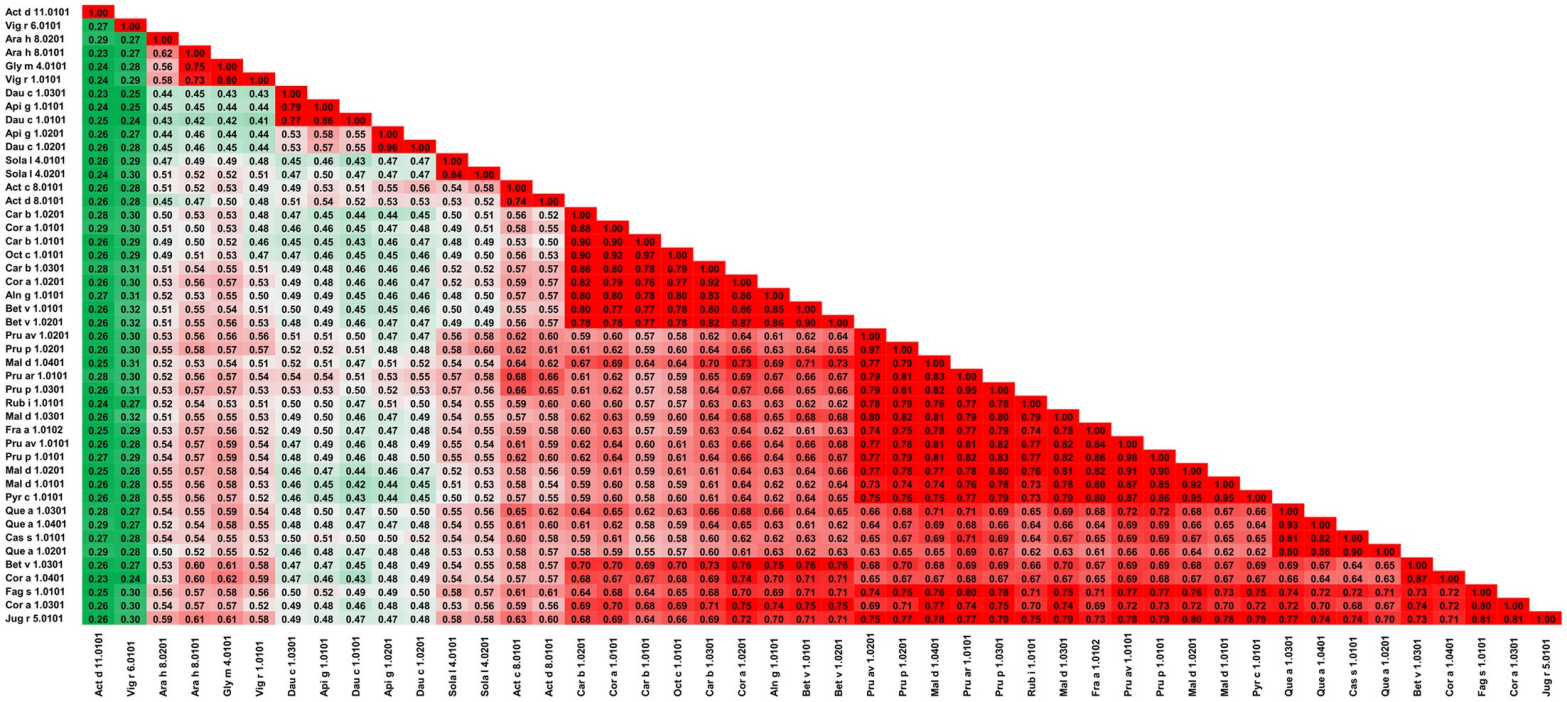
Plot showing A-RISC index values for allergen pairs from PR-10 (Bet v 1-like) family. Red color indicates a high risk of cross-reactivity, a dark green color indicates a low risk of cross-reactivity, and intermediate colors correspond to a medium risk of cross-reactivity.

Analysis of sequences of Bet v-1-like allergens allows us to group these proteins into several subgroups (Fig 25). The fist subgroup includes Act d 11.0101 and Vig r 6.0101 which are the least similar to other members of the family. The values of A-RISC indexes suggest a low or medium-low likelihood of their involvement in cross-reactive reactions. However, despite their low level of homology with Bet v 1 (Fig 25; 19% of sequence identity with Act d 11.0101 and 26% with Vig r 6.0101) both allergens are recognized by anti-Bet v 1 IgE [226, 227].

**Fig 26:**
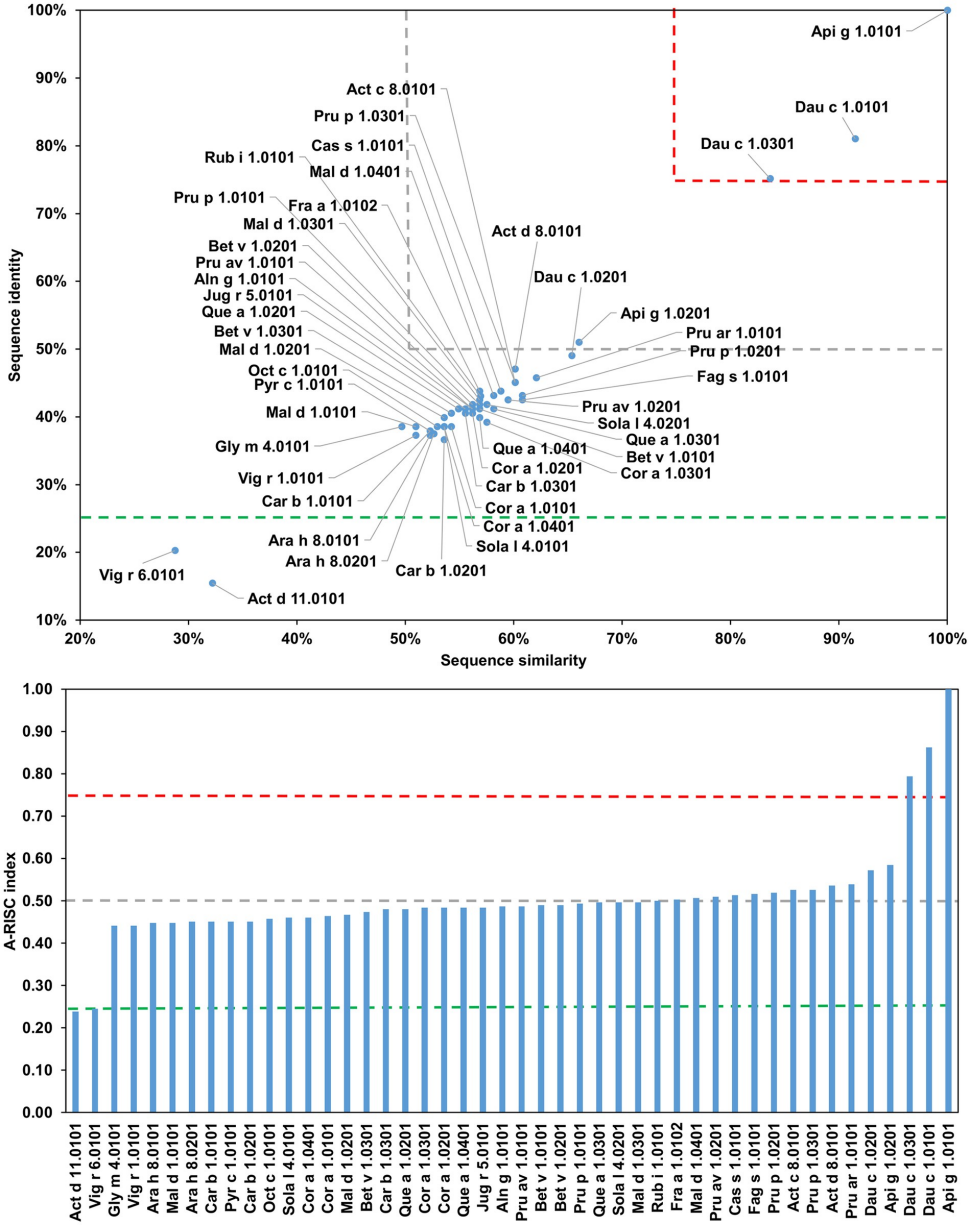
Plots showing relative homology and A-RISC indexes of allergens from PR-10 (Bet v 1-like) family. Comparison of the family members to Api g 1.0101. Red, gray, and dark green dashed lines indicate respectively 75%, 50% and 25% sequence identity and similarity, or 0.75, 0.50 and 0.25 A-RISC values.

**Fig 27:**
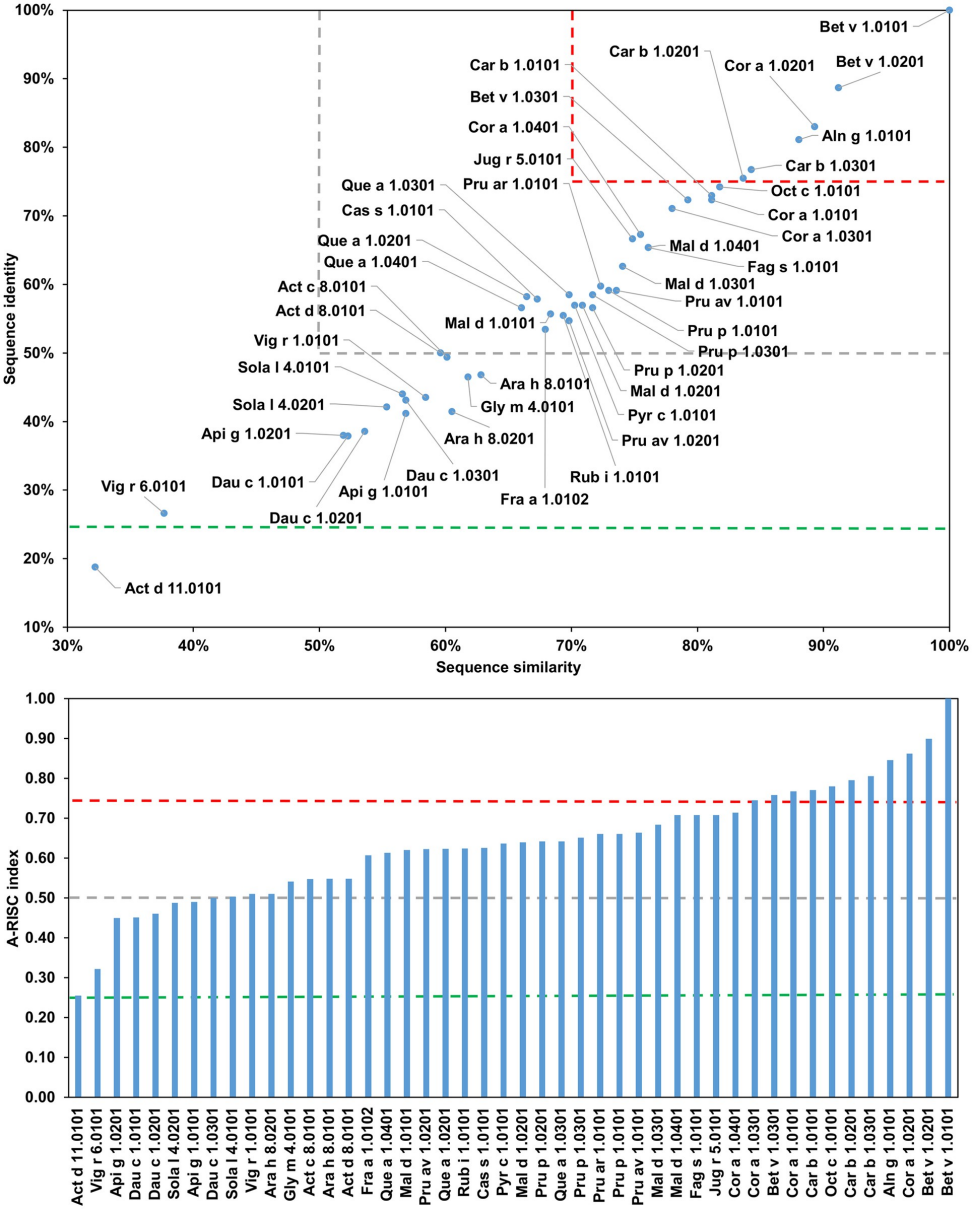
Plots showing relative homology and A-RISC indexes of allergens from PR-10 (Bet v 1-like) family. Comparison of the family members to Bet v 1.0101. Red, gray, and dark green dashed lines indicate respectively 75%, 50% and 25% sequence identity and similarity, or 0.75, 0.50 and 0.25 A-RISC values.

The second group contains Ara h 8.0101, Ara h 8.0201, Gly m 4.0101 and Vig r 1.0101. Ara h 8.0201 is less similar to other members of the subgroup for which a risk of cross-reactivity is high. These proteins are not only distinct from Act d 11.0101 and Vig r 6.0101, but also from PR-10s from celery (Api g 1) and carrot (Dau c 1; Figs 26 and 27). The allergens from subgroup II have less than 50% sequence identity with Bet v 1, but are also often reported to be cross-reactive with the birch allergen and are relevant for OAS [228–233]. The third subgroup includes Api g 1.0101, Api g 1.0201, Dau c 1.0101, Dau c 1.0201 and Dau c 1.0301. These allergens were also reported being cross-reactive with Bet v 1 [234–239]. Interestingly, Api g 1, but not Dau c 1 was shown to be able to stimulate dendritic cells from birch pollen-allergic individuals [240]. Api g 1 was also shown to be cross-reactive with Pru av 1 [241]. The fourth group includes Act c 8.0101, Act d 8.0101, Sola l 4.0101 and Sola l 4.0201. While the kiwi fruit and tomato PR-10 are not extremely similar to each other, they share a pattern of sequence identities and similarities to other members of the family.

The fifth subgroup contains Aln g 1.0101, Bet v 1.0101, Bet v 1.0201, Cor a 1 0101, Cor a 1.0201, Car b 1.0101, Car b 1.0201, Car b 1.0301, and Oct c 1.0101. The members of this subgroup are extremely similar and there is high risk of a cross-reactive reaction (A-RISC values 0.76–0.92) involving these allergens, which is consistent with reports on IgE cross-reactivity among patients [242–245].

The sixth subgroup is the most numerous and includes Fra a 1.0102, Mal d 1.0101, Mal d 1.0201, Mal d 1.0301, Mal d 1.0401, Pru ar 1.0101, Pru av 1.0101, Pru av 1.0201, Pru p 1.0101, Pru p 1.0201, Pru p 1.0301, Pyr c 1.0101, and Rub i 1.0101. Members of this group are very similar and there is a high risk of cross-reactivity among the allergens. Moreover, our analysis also indicates that this subgroup of PR-10s is associated with a high risk of cross-reactivity with Cor a 1.0301, Fag s 1.0101 and Jug r 5.0101 (Fig 25). Similar to other proteins from this family, they were also reported to be cross-reactive with Bet v 1 [228, 230, 233, 246–250].

Subgroup VII includes PR-10s from oak (Que a 1.0101, Que a 1.0201 and Que a 1.0301), as well as from chestnut (Cas a 1.0101). These similar proteins were also found to be cross-reactive with Bet v 1 [251–254]. The last subgroup includes Bet v 1.0301, Cor a 1.0301, Cor a 1.0401, Fag s 1.0101 and Jug r 5.0101. Allergens from this subgroup are not only very similar to each other, but they are also very similar to PR-10s from subgroups V-VII as well as subgroups II and IV.

## Discussion

### Sequence identity and A-RISC index

**Fig 28:**
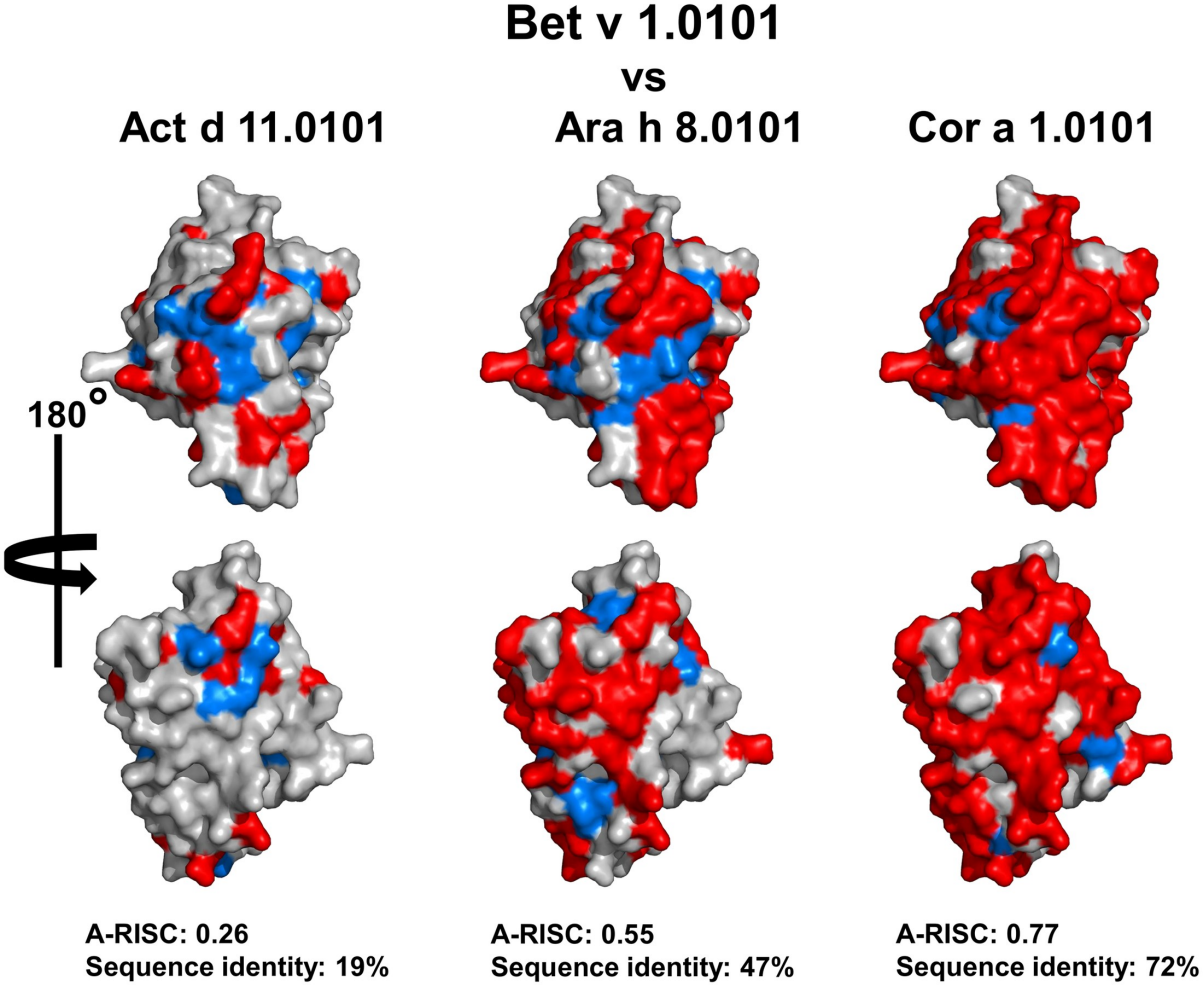
Comparison of Bet v 1.0101 with Act d 11.0101, Ara h 8.0101 and Cor a 1.0101. Identical amino acids (red) mapped on the structure of Bet v 1. Residues marked in light blue correspond to the difference between similar and identical residues.

Our analysis of the ten allergen families shows that even simple models that use analysis of protein sequences may be very helpful in understanding molecular basis of cross-reactivity. Moreover, such analysis can provide an explanation for clinically observed cross-reactivity patterns. In our opinion, analysis of sequence identities only is a minimal approach that may underestimate the risk of cross-reactivity. Introduced here, the A-RISC index is a step forward towards a better estimation of the likelihood of cross-reactivity, and when combined with structural information, illustration of the homology between pairs of allergens can be better elucidated (Figs 1 and 28). Moreover, it clearly shows why similar, but not identical residues can play a role in cross-reactivity, especially for proteins that have a relatively low sequence identity (Fig 28). For example, only consideration of identical and similar residues combination explains why a cross-reactive reaction between Bet v 1 and Act d 11 may be observed [255]. In this case, we are able to explain the formation of continuous patches on the surface of Act d 11 that may correspond to epitopes responsible for binding IgE antibodies developed against Bet v 1 [255]. Such surface patches corresponding to epitopes usually have an area of ~800 Å^2^ [11, 12]. Fig 27 also shows the importance of considering sequence similarity when comparing Act d 11.0101 and Bet v 1.0101 and shows that sequence identity alone maybe not be the best estimation of the likelihood of cross-reactivity. A similar situation is also observed for various allergens, like Aca s 2 (Fig 4A), Tyr p 2.0101 (Fig 4B) and Par j 2.0101 (Fig 22) for which sequence similarity is ~15% higher than sequence identity.

Despite various limitations, the model that we propose robustly predicts cross-reactivity patterns that were extensively elucidated experimentally. Based on our analyses, we propose to amend the original rules for allergen cross-reactivity proposed by Aalberse [28] and introduce the four following categories of cross-reactivity likelihood: high (A-RISC values ≥ 0.75), medium-high (0.75 > A-RISC ≥ 0.50), medium-low (0.50 > A-RISC ≥ 0.25) and low (A-RISC values < 0.25). Of course, these A-RISC ranges we selected are arbitrary and should be treated as general guidelines.

The sequence identity/similarity based approach works quite well for groups of proteins with members that differ significantly in protein chain lengths. For example, it is true for nsLTP-1s and nsLTP-2s, β-expansins and expansin-related proteins, or Group V and VI of grass pollen allergens. Our analysis also illustrates the striking differences between allergen families that are manifested in their internal diversity (e.g. lipocalins) or lack of such diversity (e.g. serum albumins and profilins).

**Fig 29:**
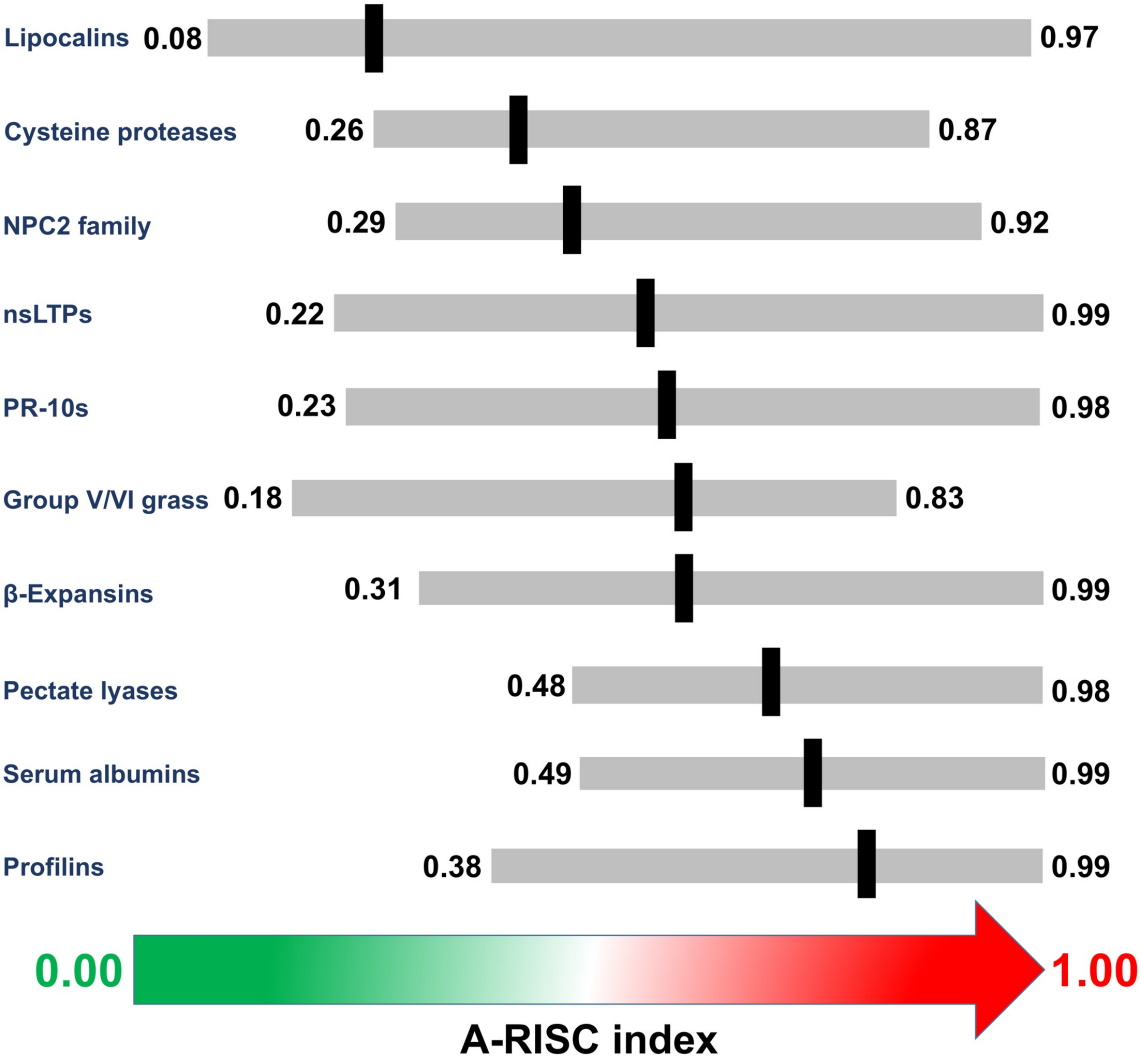
A-RISC indexes for various allergen families. Gray bars correspond to observed A-RISC ranges. The black horizontal lines indicate an average A-RISC index for a family.

Allergen families that contain at least several members may be ranked according to their propensity for cross-reactive reactions. Such a ranking may be created by calculating an average A-RISC index for the whole family. For allergen families analyzed by us (Fig 29) the propensity for cross-reactivity is as follows (average A-RISC indexes and sequence identities are reported in parenthesis): profilins (0.80, 76%), serum albumins (0.74, 71%), pectate lyases (0.64, 64%), β-expansins and expansin-related proteins (0.61, 56%), group V/VI grass pollen allergens (0.60, 57%), PR-10s (0.58, 52%), nsLTPs (0.56, 51%), NPC2 family (0.48, 41%), cysteine proteases (0.42, 37%), and lipocalins (0.26, 21%). It must be noted that this ranking corresponds to currently reported allergens, and patterns of cross-reactivity will strongly depend on allergen exposure.

### A-RISC index—Limitations of the current approach

The model we have proposed has various limitations. For example, this method is considering sequences, whilst possible posttranslational modifications of the amino acids are ignored. Posttranslational modifications resulting in the presence of hydroxyprolines, carbohydrates and other less common chemical moieties sometimes have a significant impact on the allergenic properties of proteins [215, 216, 256–258]. We also omit the fact that some of the ligands that are carried by allergens may impact the allergenicity of allergenic proteins. Moreover, even when considering only the protein sequence, we can improve the current approach by trying to map the sequence conservation on an allergen model and estimate whether the conserved residues that form patches or stretches are large enough to become epitopes (both continuous and discontinuous). As we do this, we might also need to consider that the conserved residues tend to cluster in parts of a protein important for the function and/or structure of the molecule. In addition, we did not incorporate any knowledge on IgE epitopes that was previously determined. Similarly, for allergen families like cysteine proteases, serum albums, lipocalins or profilins, which have human homologs, we did not use these sequences of human proteins to adjust our model [2].

It is also important to stress that while there is little doubt how a sequence identity is calculated, calculating sequence similarity is significantly more complex. Fig 1A demonstrates that the outcome of any sequence similarity calculation depends on the definition of similarity between amino acids. Here, definitions used by Clustal Omega and SIAS servers are completely different. The more conservative definition used in this manuscript represents chemical similarities of amino acids that is necessary for the preservation of the interaction between an allergen and antibody. Of course, the definition used here is not the only one possible, and it may be modified. For example, an improved definition of amino acid similarity that considers epitope-paratope interactions could be derived by analysis of available structures of protein-antibodies complexes that are deposited to the Protein Data Bank (PDB) [259]. Unfortunately, PDB contains mostly complexes between antigens and IgG derived antibodies fragments, which in the case of allergens most often are complexes with mouse IgGs [260–263], and therefore, this analysis may not fully represent amino acids that are preferred in interactions with IgEs. Furthermore, one should remember that not only amino acids are found at the interfaces of proteinous antigens and antibodies. Interactions between antigens and antibodies may be mediated by water molecules, carbohydrates and various ions that are trapped between the antigen and antibody [260, 261].

### Allergen families and nomenclature of allergens

Many registered allergens were omitted in our calculations because they do not have a complete sequence determined. This highlights the need for researchers to identify the sequences of allergens, as well as the need for an allergen’s complete sequence to be determined for its registration by the WHO/IUIS Allergen Nomenclature Sub-committee. Moreover, a significant fraction of available literature on experimental determination of sensitization and/or cross-reactivity is based on studies that used extracts. Such publications provide useful information on the cross-reactivity of proteins originating from various sources but do not provide enough detail to determine which allergens or isoallergens are responsible for cross-reactivity. Our studies strongly highlight the need for the standardization of allergen extracts. It is especially important in situations when there are several allergens from the same protein family that originate from a single source. In such cases, these proteins will have similar molecular weights and other properties, which in turn may easily lead to their misidentification if only simple methods like SDS-PAGE or blotting with pooled patient sera are used for their discrimination. For example, one may imagine a situation in which *D*. *farinae* allergens are studied and the Western blot indicates IgE binding to a protein (or proteins) with molecular weight of ~14 kDa. In fact, this band may correspond to Der f 2, Der f 22 or Der f 35, as well as a mixture of any of these allergens. Similar situations could happen with lipocalins originating from dog (Can f 1, Can f 2, Can f 4 and Can f 6), nsLTPs originating from tomato (Sola l 3, Sola l 6 and Sola l 7) or peanut (Ara h 9, Ara h 16 and Ara h 17). In all these cases, the difference in molecular weight will be in the 1–2 kDa range, therefore making it easy to misrecognize the proteins. The discrimination between these allergens may be even more complicated when various posttranslational modifications are considered, as well as the presence of various isoallergens.

The fact that allergenic proteins belong to a relatively small group of protein families is extremely important for understanding the molecular bases of allergic diseases. This also helps to build the allergen nomenclature that is clearly related to the concept of protein families [1]. It is also likely that the current guidelines related to division of allergens into isoallergens (sequence identity >67%) and variants of isoallergens (sequence identity >90%) will have to be reevaluated once more data on various isoallergens will be accumulated. In addition, the concept of dividing allergens into major and minor should be made more precise, as it currently refers to the whole set of similar molecules present in an allergen source, rather than a well-defined molecular entity for which the relative abundance is known.

While the presence of isoallergens and their variants is challenging for standardization of extracts [264] and allergy diagnostics, it is also fascinating from the perspective of molecular allergology and the origin of an allergic disease, because despite their similarities, these molecules can display different behaviors when in contact with the human immune system [102, 265, 266]. This difference in behavior may be key to understanding the allergy origin and key in the design of immunotherapy.

### Applications of the A-RISC index

Currently, many diagnostic tools that can be used to detect IgE binding to individual allergenic proteins are available. Tests including multiplex platforms provide a great body of information concerning IgE binding to individual allergens. Understanding the cross-reactivity of individual allergenic proteins may help in explaining the results of allergy testing [257, 267]. Therefore, we are convinced that the A-RISC index can be used in the interpretation of results originating from component resolved allergy diagnostics. Considering the problem of allergen cross-reactivity that is often encountered in allergy diagnosis, analysis of IgE reactivity to individual allergens may allow for better selection of true sensitizers in patients allergic to multiple allergens. In addition, it may help to design appropriate composition of vaccines and immunotherapeutics. Several reports provide evidence of how individual allergens present in vaccines may induce a protective immune response to cross-reactive allergens [268–271]. Moreover, the idea of analyzing allergens in the context of protein families, like in the calculation of A-RISC indexes, should provide better avoidance guidelines, as they may include not only information on molecules that are suspected to be the primary sensitizers, but also provide a list of allergens (and their sources) that may be involved in various cross- reactions. Furthermore, the sequence derived guidelines, for example based on A-RISC indexes, are able to provide a general categorization of the cross-reactivity likelihood.

The analysis of allergen families also shows that due to their internal diversity, or lack thereof, proteins will be related to each other with completely different patterns of possible cross-reactivities. For example, allergenic profilins and serum albumins will be most likely associated with high likelihood of cross-reactivity, while it may be not true for other allergen families. It is also worth it to highlight that even in a particular allergen family, one can delineate several subgroups of proteins that are more likely to be responsible for cross-reactivity. Such a division may be simple, like in the case of pectate lyases that have two distinct subgroups, or more complicated like in the cases of lipocalins, β-expansins, nsLTPs and PR-10s that have many members.

Comparison of an allergen with other family members may be especially informative assuming that the first one is known to be the primary sensitizer. Presented herein, plots showing relative sequence similarities and identities, as well as A-RISC indexes can be grouped into three general categories. The first category corresponds to the situation that is observed for example when one assumes that Der p 2 (Fig 4A), Jun a 1 (Fig 10B) or Can f 1 (Fig 12) are primary sensitizers. In these cases, a clear division between family members is observed, and it can facilitate prediction of cross-reactivity patterns. The second category corresponds to a situation observed for Phl p 1 (Fig 16), Pru p 3 (Fig 22) and Bet v 1 (Fig 27), in which the other members of the family are linearly and consistently distributed over the whole range of sequence similarity and identity. This category is especially interesting, as these three allergens are implicated in many cross-reactive reactions that are clinically relevant. Finally, the third category corresponds to distributions observed for Blo t 2 (Fig 4B) and Equ c 1 (Fig 13) that may be treated as a combination of the first and second categories. The categorization of the relative patterns of sequence identities and similarities may further facilitate prediction of the likelihood of cross-reactivity including allergenic proteins not yet characterized.

## Supporting information

S1 Table Allergens from cysteine protease family. (XLSX)

S2 Table Allergens from NCP2 family of proteins. (XLSX)

S3 Table Allergens from serum albumin family of proteins. (XLSX)

S4 Table Allergens from pectate lyase family of proteins. (XLSX)

S5 Table Allergens from lipocalin family of proteins. (XLSX)

S6 Table β-Expansisns and expansin-related proteins. (XLSX)

S7 Table Group V/VI grass pollen allergens. (XLSX)

S8 Table Allergens from profilin family of proteins. (XLSX)

S9 Table Allergens from nsLTP family of proteins. (XLSX)

S10 Table Allergens from PR-10 (Bet v 1-like) family of proteins. (XLSX)
